# Heterosubtypic Immunity to Influenza A Virus Infections in Mallards May Explain Existence of Multiple Virus Subtypes

**DOI:** 10.1371/journal.ppat.1003443

**Published:** 2013-06-20

**Authors:** Neus Latorre-Margalef, Vladimir Grosbois, John Wahlgren, Vincent J. Munster, Conny Tolf, Ron A. M. Fouchier, Albert D. M. E. Osterhaus, Björn Olsen, Jonas Waldenström

**Affiliations:** 1 Centre for Ecology and Evolution in Microbial Model Systems (EEMiS), Linnæus University, Kalmar, Sweden; 2 International Research Center in Agriculture for Development (CIRAD)–UPR AGIRs, Animal and Integrate Risk Management, Campus International de Baillarguet, Montpellier, France; 3 Karolinska Institutet, Department of Microbiology, Tumor and Cell Biology (MTC), Stockholm, Sweden; 4 Department of Virology, Erasmus Medical Center, Rotterdam, The Netherlands; 5 Laboratory of Virology, Division of Intramural Research, National Institute of Allergy and Infectious Diseases, National Institutes of Health, Hamilton, Montana, United States of America; 6 Section of Infectious Diseases, Department of Medical Sciences, Uppsala University, Uppsala, Sweden; Imperial College London, United Kingdom

## Abstract

Wild birds, particularly duck species, are the main reservoir of influenza A virus (IAV) in nature. However, knowledge of IAV infection dynamics in the wild bird reservoir, and the development of immune responses, are essentially absent. Importantly, a detailed understanding of how subtype diversity is generated and maintained is lacking. To address this, 18,679 samples from 7728 Mallard ducks captured between 2002 and 2009 at a single stopover site in Sweden were screened for IAV infections, and the resulting 1081 virus isolates were analyzed for patterns of immunity. We found support for development of homosubtypic hemagglutinin (HA) immunity during the peak of IAV infections in the fall. Moreover, re-infections with the same HA subtype and related prevalent HA subtypes were uncommon, suggesting the development of natural homosubtypic and heterosubtypic immunity (*p*-value = 0.02). Heterosubtypic immunity followed phylogenetic relatedness of HA subtypes, both at the level of HA clades (*p*-value = 0.04) and the level of HA groups (*p*-value = 0.05). In contrast, infection patterns did not support specific immunity for neuraminidase (NA) subtypes. For the H1 and H3 Clades, heterosubtypic immunity showed a clear temporal pattern and we estimated within-clade immunity to last at least 30 days. The strength and duration of heterosubtypic immunity has important implications for transmission dynamics of IAV in the natural reservoir, where immune escape and disruptive selection may increase HA antigenic variation and explain IAV subtype diversity.

## Author Summary

Influenza A viruses (IAV) infect a range of hosts, with the largest diversity being found in waterfowl, particularly dabbling ducks. In these hosts, IAV causes only mild disease, while viruses that infect other hosts, such as poultry, horses or humans, can cause fatal infections. In fact, all known pandemic flu viruses have contained gene segments that originated in the wild bird reservoir. We sampled a wild population of Mallards over eight seasons and characterized infection histories in 7728 birds. For hemagglutinin (HA) the subtype recoveries indicated that once a Mallard has been infected, re-infection with the same HA subtype is uncommon within the next month, clearly indicating homosubtypic immunity. Moreover, we found evidence for natural heterosubtypic immunity, where phylogenetically related HA subtypes at clade and group levels were less common in re-infections than expected. On the contrary no specific patterns of immunity was found for neuraminidase subtypes. IAVs exist in numerous antigenic subtypes that co-circulate. The strength of heterosubtypic immunity in natural infections provides evidence that HA subtypes compete over hosts and that immune escape may result in positive selection for HA antigenic variation in the virus, and thus explain IAV subtype diversity.

## Introduction

Influenza A viruses (IAVs) infect many different avian and mammalian hosts, including humans [1], [2]. Among avian hosts, the largest viral genetic diversity and highest prevalence are found in species associated with wetlands [2]. Consequently, waterfowl, particularly dabbling ducks but also other anatids, shorebirds and gulls are considered as the main reservoirs for IAV [2]. Avian and mammalian influenza, including seasonal and pandemic flu in humans, are epidemiologically linked. The common view is that the genetic variation occurring in the wild bird reservoir can be seeded into other host species through *de novo* introductions, or through reassortment processes in animals permissive to both avian and mammalian-adapted viruses [3]. Given that the highest levels of IAV genetic variation are found in wild waterfowl, it is critical to understand evolution of the virus in these hosts.

**Figure 1:**
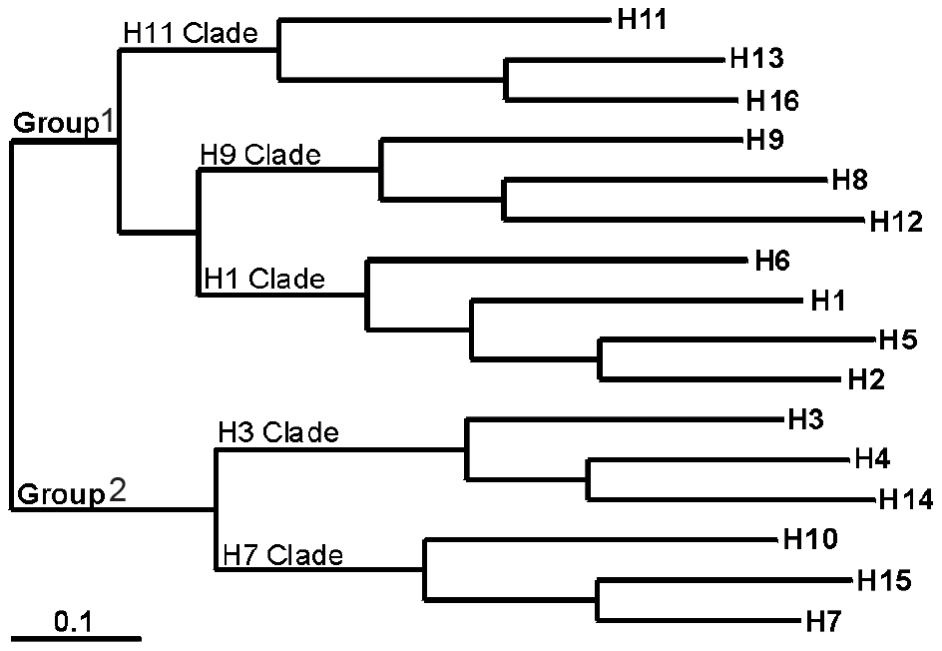
DNA maximum-likelihood trees of the HA segment. illustrating relationships between IAV hemagglutinin subtypes. Branch lengths represent the number of nucleotide substitutions between close HA subtypes (scale bars represent 10% of nucleotide substitutions). Adapted from Fouchier et al. 2005.

Diversification and evolution of IAV genotypes is primarily shaped via mutation and reassortment. At least in part, genotype evolution may be driven by host responses to infection. The hemagglutinin (HA) protein is the most abundant surface glycoprotein on the IAV membrane, responsible for the attachment and fusion of the virion with the host cell at the start of infection. The epitopes that interact with antibodies are a major target for the host immune response, hence HA genotypes should be subjected to strong selection for immune escape [4], [5]. Neuraminidase (NA) is the second most abundant membrane protein which, during infection, cleaves the terminal sialic acid residues from the newly formed virions and host cell receptors [1]. NA is also an important target for the host immune system, but NA antibodies do not neutralize IAV [4]. Currently, 16 HA and 9 NA antigenic variants are recognized in birds, which can theoretically be combined into 144 different HA/NA subtype combinations [6]. The HA phylogeny reflects divergence that occurred at different times represented as relatedness at higher-order clustering of subtypes [6], [7]. For example, the H1, H2, H5 and H6 subtypes belong to the H1 Clade, while H7, H10 and H15 belong to the H7 Clade (Figure 1). At an even higher level, the different clades can be classified into Group 1 (H11, H9 and H1 Clades) and Group 2 viruses (H3 and H7 Clades). Within HA clades, subtypes have 62–68% amino acid similarities, while subtypes from the two different HA groups have approximately 40% amino acid similarity.

Antagonistic co-evolution in a sympatric model of circulating IAVs predicts that a change in the virus will modulate a change in the host immune system and result in phylogenetic branching of viruses genotypes into discrete antigenic IAV subtypes, with little or non-overlapping antigenic spaces [8]. Thus, a central question is to understand how changes in cross-protective HA or NA immunity will translate into viral fitness effects and influence IAV diversity and evolution [7].

To this end, most studies have relied on experimental infections or vaccinations to study the development of immunity in birds [9]–[12]. Generally, waterfowl are permissive to experimental infection with low pathogenic avian influenza (LPAI). Naïve ducks (usually domestic variants of the Mallard *Anas platyrhyncos*) produce a primary infection which lasts from 4 to 21 days [10], [13], [14]. Infection with a particular virus subtype induces homosubtypic immunity, at least in the short-term [10], [12], and is correlated with specific antibody production [10], [12], [15]. Interestingly, there are also indications that infection with one subtype could induce partial, or complete, immunity to heterosubtypic re-infections [11], [12]. The degree of homo- and heterosubtypic immunity in re-infections has strong relevance for IAV infection dynamics and subtype evolution. So far, homo- and heterosubtypic immunity has mainly been studied in the context of epidemiology of highly pathogenic avian influenza (HPAI), in particular for the HPAI H5N1 virus that has circulated among poultry since 1997 [16]. Ducks and geese pre-exposed to LPAI have reduced severity of HPAI H5N1 induced symptoms, and lower mortality, after experimental infection [9], [17]–[19]. Furthermore, homosubtypic HA vaccination of poultry against HPAI H5, HPAI H7 and LPAI H9 is used for virus control and eradication in endemic areas and is usually associated with good protection [20]. Vaccines composed of a homologous HA and a heterologous NA to the epizootic virus are a useful tool for control of HPAI in poultry [21], [22]. There is extensive interest in finding universal vaccines for humans, and attempts to exploit heterosubtypic immunity (to confer protection against several subtypes) are currently underway [23]–[25]. Contrary to poultry or humans, there is only scant information on IAV immunology and development of immune responses in wild bird reservoirs [15], [26], [27]. This is unfortunate, since the degree of individual and herd immunity will influence disease dynamics by affecting the number of susceptible hosts, and thus the strength of selection on IAV for immune escape. Furthermore, if the degree of heterosubtypic immunity varies in reciprocal strength between two subtypes it could select for one subtype over the other, via inter-subtype competition over hosts [28]. During recent years, large-scale sampling for IAV in wild waterfowl populations have been conducted in several parts of the world. However, most studies have dealt with prevalence/seroprevalence at the population level, rather than at the individual scale by repeated sampling of the same individuals. In depth studies on development of immunity rely on repeated sampling and construction of individual infection histories. While some studies have been conducted using sentinel ducks [29], [30] it is preferable to use wild individuals displaying their full range of natural behaviour. In the present study, we analyzed IAV subtype infection patterns in a well-characterized wild migratory Mallard population at an important stopover site in Southern Sweden [31], [32]. Through capture and recapture of birds during their fall stopover, we acquired unique individual infection histories of wild birds. These allowed us to test whether relatedness of HA or NA subtypes at the first detected infection influenced the likelihood of later re-infection with homo- and heterosubtypic virus subtypes.

## Results

### General description

From 2002 to 2009, a total of 7728 individual Mallards (4988 males, 2660 females, 80 unsexed) were captured and sampled at Ottenby Bird Observatory, SE Sweden. Of the birds trapped, 3856 were juveniles, 2451 were adults (*i.e.* birds fledged the previous year or earlier) and 1421 could not be certainly aged by plumage criteria, and were left as unaged. Approximately one third of the birds were retrapped at least once at Ottenby during the same season (*n* = 2439), the maximum number of recapture occasions was 61 for a single individual (range from 1–61 times in different birds), giving a total of 18679 samples analyzed for IAV. The average stopover time, measured as the difference between first and last recapture in the fall, was 17.1 days (SD 0.5, years 2002–07). In total, 2451 samples were positive for IAV (13.1% overall IAV prevalence), as determined by real-time reverse transcriptase PCR assay (RRT-PCR). For RRT-PCR positive birds, the maximum number of infection positive days in a single individual within the season was 13 occasions (range of 1–13 amongst individuals). RRT-PCR positive samples were propagated in hen eggs, yielding a total of 1081 virus isolates (44% isolation success). The maximum number of isolates from a single individual was 9. Overall, 10.6% of the Mallards (822 individuals) had at least one infection where an isolate was retrieved. In 98 cases, these birds had the same HA/NA subtypes in two or more virus isolates. The 95% confidence interval estimation of the minimal duration of virus shedding was [2.66–3.36] days. The minimal duration of shedding, in days, was modelled using generalized linear models and the significance of the explanatory variables was assessed using likelihood ratio tests (LRT). That did not vary across months (July to December; df = 5; LRT *p*-value = 0.88), years (2002–09; df = 7; LRT *p*-value = 0.99), HA subtypes (H1–H8, H10–H12; df = 10; LRT *p*-value = 0.99) or as a function of number of previous detected infections (1 to 3 infections; df = 1; LRT *p*-value = 0.97). Shedding was intermittent or discontinuous in 11 individuals, similar to previous studies [12], [33].

### HA subtypes in consecutive infections

**Figure 2:**
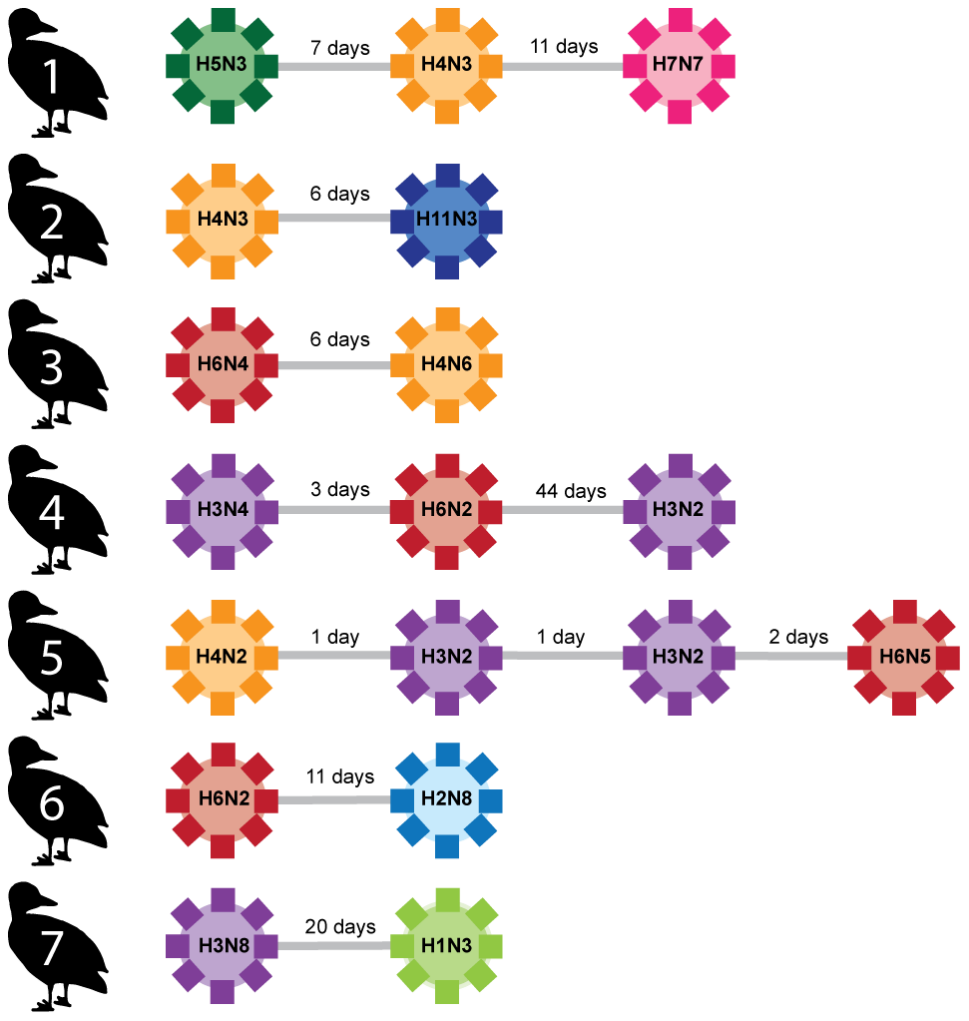
Examples of consecutive IAV infections in seven individuals. The subtypes of detected viruses, as well as time between detections, are given.

In 104 individuals, at least two separate infections were detected within a fall season – the data from these individuals were used to determine patterns of homosubtypic and heterosubtypic immunity (examples in Figure 2). The time between successful isolation of two different viruses (*i.e.* different subtypes or independent infection events) varied from 1–49 days in different individuals, with a mean of 8.38 days and a median of 3 days (*n* transitions = 142, SD 9.69; Figure S1). These data allowed the testing of two hypotheses: (1) that an infection with a specific HA or NA subtype would confer protection against future homosubtypic infections, and (2) that an infection with a specific HA or NA subtype would confer protection against future heterosubtypic infections of phylogenetically closely related subtypes (for example, infection with a Clade 1 subtype would protect against infection with other Clade 1 subtypes).

Using the whole dataset (Table S1) and disregarding time between detected infections, we tested the null hypothesis that the subtypes of successive virus detections were independent. In this case, H_0_ could not be rejected (11*12 contingency table (hereafter CT), *n* individuals = 104, *n* transitions = 142, median of the Monte Carlo Fisher's exact test *p*-value over randomly generated subsamples where each individual contributed with a single detection pair = 0.58 (hereafter median MC Fisher's *p*-value, see Method section for details)) suggesting independence between successive infections in single individuals. The independence hypothesis could also not be rejected when contingency tables were built using HA relatedness at group or clade levels while still ignoring time between infections (2*2 CT for groups, 5*5 CT for clades, sample size as above, median Fisher exact *p*-value over randomly generated independent subsamples = 0.11 (hereafter median Fisher's *p*-value) and median MC Fisher exact *p*-value = 0.56 for groups and for clades, respectively; Tables S2 and S3).

To refine the analyses, we introduced time between infections as a variable categorized as either short lag (≤6 days between isolation) or long lag (≥7 days between isolation). This cut-off was based on the estimated duration of shedding from natural infections [31] and experimental infections [14] and was used in order to disentangle new infections from old infections. This also accounted for the time lag between first contact with the virus and the development of immune responses, thus we expected to detect immunological resistance patterns in the long lag category as a result of induced protective immunity. For short lag pairs, independence between HA subtype at first detection and at second detection could not be rejected (11*12 CT, *n* individuals = 70, *n* transitions = 84, median MC Fisher's *p*-value = 0.18; Table S4). The independence hypothesis could also not be rejected for short lag detection pairs when contingency tables were built using HA relatedness at group or clade levels (2*2 CT for groups, 5*5 CT for clades, sample size as above, median Fisher's *p*-value = 0.24 and 0.45 for groups and clades, respectively; Tables S5 and S6). For long lag pairs, the independence hypothesis test was close to significance at the subtype level (11*12 CT, *n* individuals = 44, *n* transitions = 58, median MC Fisher's *p*-value = 0.06; Table S7 and S8), non-significant at the clade level (5*4 CT, sample size as above, median Fisher's *p*-value = 0.28; Table S9) and close to significance at the group level (2*2 CT, sample size as above, median Fisher's *p*-value = 0.07; Table S9). It has to be noted that the independence tests presented above are quite conservative with regards to the development of immunity, because they consider any type of departure from independence as the alternative hypothesis, whereas immunity should result in specific patterns of departure from independence where re-infections by a virus of the same subtype or by a phylogenetically related subtype are observed less frequently than expected under the null independence hypothesis. Indeed, examining the standardized Pearson's residuals (*r_ij_*) in the long lag contingency tables at the subtype, clade and group levels revealed such patterns of departure from independence. At the subtype level (Table S8), negative mean standardized Pearson's residuals were observed for cells corresponding to same subtype (mean *r_ij_* = −0.55) or related subtypes from the same clade (mean *r_ij_* = −0.49), and positive mean residuals for cells corresponding to unrelated subtype pairs (mean *r_ij_* = 0.15). At the clade level the residuals were negative for re-infections with the same clade (mean *r_ij_* = −1.23) and positive for re-infections from a different clade (mean *r_ij_* = 0.28), and similar patterns were seen also at the HA group level (mean *r_i_* = −1.99 for same group cells and mean *r_ij_* = 1.99 for different groups cells; Table S9).

**Figure 3:**
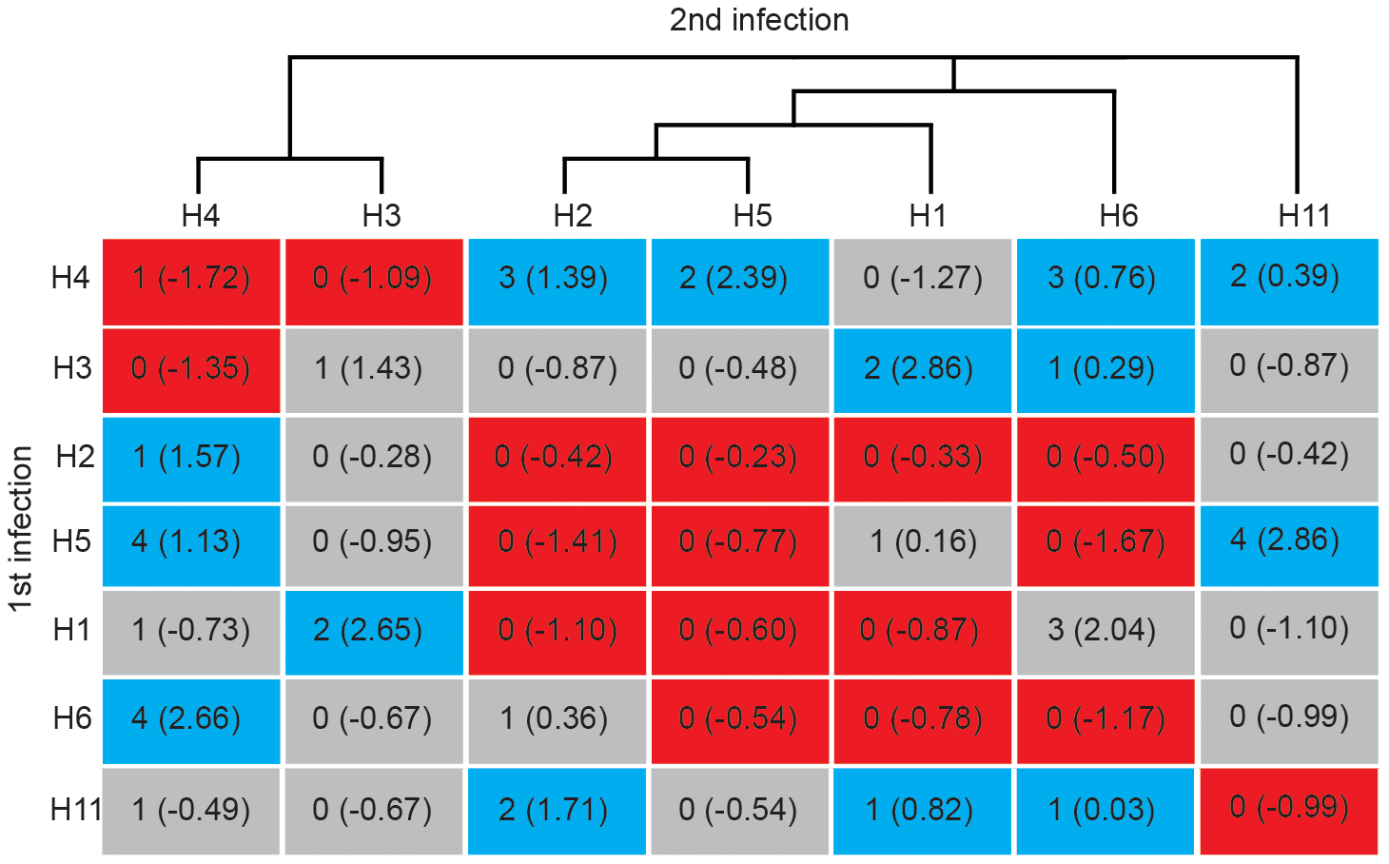
Contingency table for HA re-infections with the IAV subtypes most frequently isolated. Rows represent the HA subtype in the first detected infection and columns the HA from an infection retrieved ≥7 days later. The number in each box represents the number of cases, together with standardized Pearson's residuals in brackets. Red boxes show negative values and represent a deficiency of cases, blue boxes show positive values and an overrepresentation of cases, and grey denote cases that do not depart from expected values. The dendrogram on top illustrates HA subtype phylogenetic relatedness.

Moreover, some subtypes included in the contingency tables were rarely detected in the wild population of Mallards despite extensive sampling and are thus likely to circulate only intermittently in the study population. A consequence of intermittent circulation could be that during some periods, or years of the study, the exposure of mallards to the rare subtypes would be null, and thus infection by these subtypes impossible. The observed contingency table frequency patterns could then be partly driven by the presence/absence of the rarer subtypes in the pool of circulating IAV subtypes, and not only by development of immune responses against certain subtypes. In order to address this issue, we run the analyses with only the HA subtypes that were detected all years in the sampled population and included them as 7 discrete categories (H1, H2, H3, H4, H5, H6 and H11) in the test of independence for the long lag infection pairs (Figure 3). These common subtypes were frequently isolated and likely co-circulated every year in the study population. Thus, a deficiency of re-infections with specific subtypes on the long lag can therefore be interpreted as a consequence of immunity. There was a significant dependence between HA subtype of first infection and re-infections in the long lag for the common subtypes (7*7 CT, *n* individuals = 32, *n* transitions = 41, median MC Fisher's *p*-value = 0.02; Figures 3 and S2, Table S8). We examined the specific patterns of re-infection and direction of the departure from independence for the common subtypes using standardized Pearson's residuals (Figure 3). We expected low frequencies for homosubtypic HA re-infections (diagonal cells in the contingency table for all transitions, Figure 3), and indeed the observed frequencies of re-infections with the same HA subtype were lower than expected for all HA variants (mean *r_ij_* = −0.64), except for H3. Two homosubtypic re-infections were detected: H3N4 initial infection, followed by a H3N2 re-infection, and H4N6 as both initial and re-infection subtype. The cases of homosubtypic re-infections occurred 47 and 49 days after the first detected infection, respectively. In both cases, the HAs from the primary and the secondary infections showed a 98% sequence similarity (GenBank accession number: KC342608 and KC342612, KC342624 and KC342628). A striking finding was that phylogenetically related subtypes (*i.e.* H1, H2, H5 and H6 in the H1 Clade) did not cause re-infections in individuals (mean *r_ij_* = −0.50) even if these subtypes co-circulated in the population. Conversely, less related subtypes (such as H4 and H2, H4 and H5 or H4 and H6), caused re-infection more often than expected (mean *r_ij_* = 0.40; Figure 3 and Table S8).

In addition, consecutive infections with a HA subtype that belonged to the same phylogenetic clade were generally less frequent than expected (mean *r_ij_* = −2.20), whereas consecutive infections with distinct HA subtypes that belonged to separate clades were more frequent than expected (mean *r_ij_* = 1.10) (3*3 CT, *n* individuals = 32, *n* transitions = 41, median Fisher's *p*-value = 0.04; Table 1 and S9, Figure S2B). Similar effects were seen at the HA group level (2*2 CT, sample size as above, median Fisher's *p*-value = 0.05; Table 2 and S9, Figure S2B). These patterns suggest that phylogenetically related HA subtypes induce a significant level of heterosubtypic immunity.

**Table 1:** Contingency table for HA re-infections with common HA clades.

	2^nd^ infection		
1^st^ infection	H1 Clade	H3 Clade	H11 Clade
H1 Clade	5 (−3.28)	12 (0.82)	4 (2.8)
H3 Clade	11 (1.49)	2 (−0.99)	2 (−0.82)
H11 Clade	4 (2.39)	1 (−0.18)	0 (−2.35)

Rows represent the HA clade in the first detected infection and columns the HA clade from an infection retrieved ≥7 days later (*i.e.* long lag). The number in each cell represents the number of cases, the standardized Pearson's residuals are provided in brackets.

**Table 2:** Contingency table for HA re-infections with HA groups.

	2^nd^ infection	
1^st^ infection	Group 1	Group 2
Group 1	13 (−1.99)	13 (1.99)
Group 2	13 (1.993)	2 (−1.99)

Rows represent the HA group in the first detected infection and columns the HA group from an infection retrieved ≥7 days later (*i.e.* long lag). The number in each cell represents the number of cases, the standardized Pearson's residuals are provided in brackets.

As noted above, the restriction to the 7 most common HA subtypes was based on epidemiological information from the study population. However, in order to determine the extent of the observed dependency we performed additional analyses where we started with the three most common subtypes (as the 2 most common generated a 2*1 table) and then subsequently included subtypes in the analyses based on the frequency of detection in the population (Table S8). There was a consistent pattern for long lag re-infection pairs with significant dependency (*i.e. p*-value ≤0.05) observed in 5 out of the 9 possible tables (Table S8). Furthermore, in the 4 remaining tables, the *p*-value was close to significant in three tables (*i.e. p*-value ≤0.10) when including 4, 6 or all subtypes in the analyses, but not in the table with only three subtypes included. In addition, the mean of the standardized Pearson's residuals were generally negative for cells corresponding to infections by same subtype, or for cells corresponding to infections by subtypes belonging to the same clade (Table S8). A similar analysis at the level of clades showed a significant dependence for the 2 and 3 most common clades (which represented the 7 most prevalent subtypes found every year), but non-significant departures from independence when rarer clades were included, although the patterns of the residuals were still compatible with the immunity hypothesis (Table S9).

When similar full explorations were performed either for the whole dataset independently of the time between infections, or for the short lag only, the results showed no departure from independence, or any specific patterns between infections at the level of subtype (Tables S10 and S11) or clade (Tables S12 and S13).

### Temporal variation of HA clades detection probabilities

**Figure 4:**
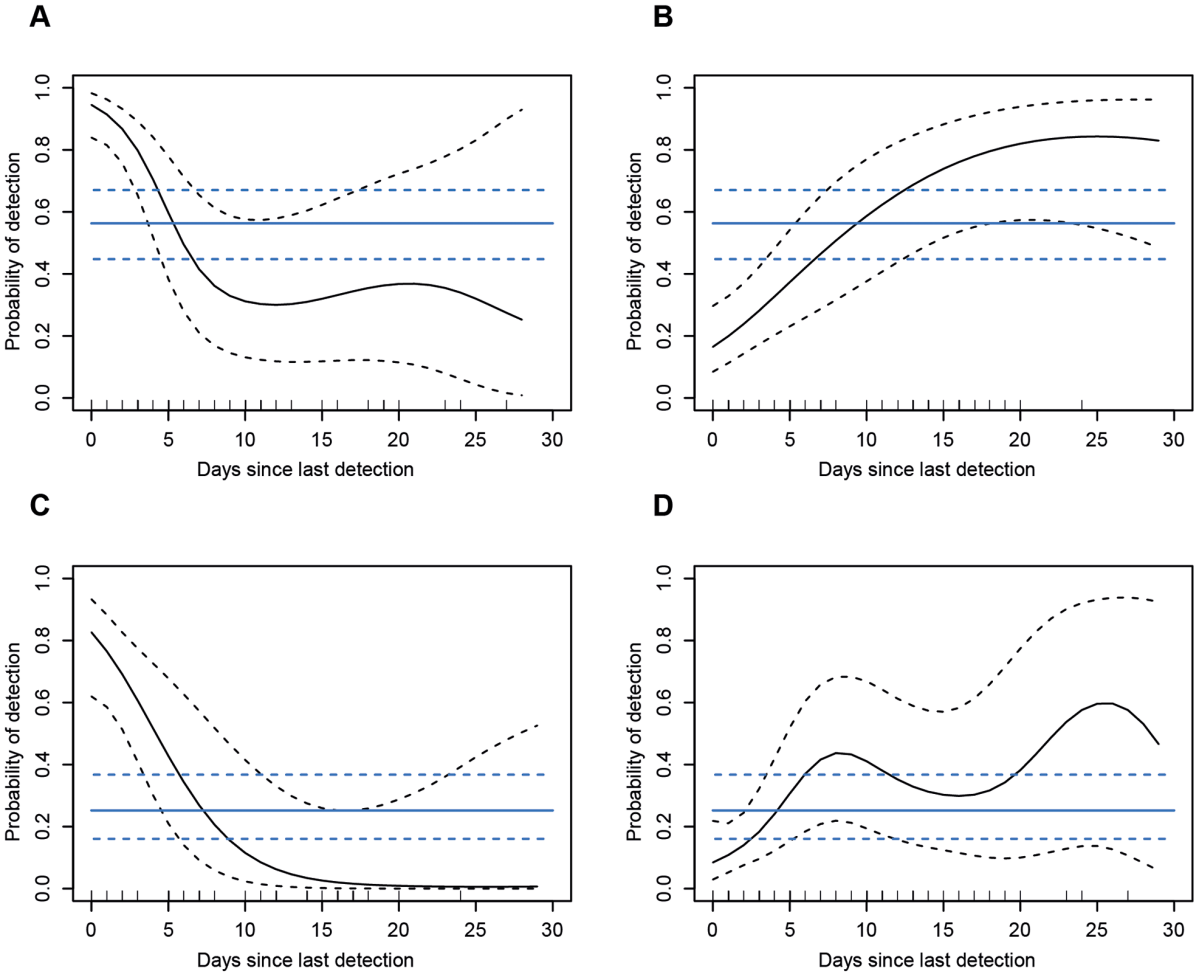
IAV detection probability for H1 and H3 Clades as a function of time and previously detected infections. The x-axis represents time in days since first detection of a virus. The y-axis depicts the probability of detection. Black continuous line represents the change over time in probability (95% CI with dashed lines). The horizontal blue line is the probability of detection of an infection with a virus from a specific clade for naïve birds (95% CI with dashed lines). The distribution of data points is presented as rug plots along the x-axis. Detection probabilities for H1 (A) and H3 (C) Clades for individuals that have previously experienced an infection with a virus from the same clade. Detection probabilities for H1 (B) and H3 (D) Clades for individuals that have previously experienced an infection with a virus from another clade.

The time component of the heterosubtypic immunity within the specific HA clades was investigated by modeling the detection probabilities of viruses belonging to the same clade as a function of time since previous detected infection (specifically based on individual infection histories). For this analysis, all HA records of infections from recaptured Mallards (*n* individuals = 585) were considered. For most HA clades, the model with strongest support included the effect of previous infection history (both for viruses belonging to the same and different clades), as well as year and seasonal effects (Table S14). Seasonal variation of relative prevalence for the H3 Clade showed that these viruses were dominant at the beginning of the fall, while viruses from the H1 Clade were dominant towards the end of the season (Figure S3). For birds infected with either a H1 or a H3 Clade virus, the probability of acquiring a new infection with a virus from the same clade decreased as a function of time (Figure 4A and 4C). Initially, the probability curves were higher than the probability of a naïve Mallard acquiring an infection from the same clade, but after approximately 7 days when infections are cleared, the probability curves dropped below the probability of a naïve Mallard acquiring an infection. This strongly suggests that the developed immune responses induce protection. It should further be noted that this effect lasted for at least 30 days (Figure 4A and 4C). Conversely, the probability of infection with a virus belonging to a different HA clade than that of the initial infection increased over time (Figure 4B and 4D). This probability was initially low and increased above that of a naïve Mallard as time progressed, during which immune responses against the clade causing the primary infection were established (Figure 4B and 4D).

Similar patterns of variation in detection probabilities in relation to previous infection history were evident in the H7, H9, and H11 Clades (Figure S4), despite the low prevalence rates of these subtypes (Figure S5) compared to those of the H1 and H3 Clades (Figure S3).

### NA subtypes in consecutive infections

Employing the same approach as described for the HA, we explored the patterns of re-infections for the NA subtypes. In order to determine patterns of re-infections for different NAs, We constructed a contingency table with the most common NA subtypes (N1, N2, N3 and N6; Table 3) similar to the analyses for HA above. No departures from the hypothesis of non-restricted transitions from primary to secondary infections with viruses of different NA subtypes were observed, regardless of time between infections (4*4 CT, *n* individuals = 48, *n* transitions = 61, median Fisher's *p*-value = 0.59). Furthermore, no effect was observed in the short time lag (≤6 days between isolation, 4*4 CT, *n* individuals = 35, *n* transitions = 40, median Fisher's *p*-value = 0.57) or in the long time lag (≥7 days between isolation, 4*4 CT, *n* individuals = 16, *n* transitions = 21, median Fisher's *p*-value = 0.87), indicating that the NA subtypes of initial infection and re-infection were independent.

**Table 3:** Contingency table for NA re-infections.

	2^nd^ infection			
1^st^ infection	N1	N2	N3	N6
N1	1	5	1	6
N2	3	5	2	7
N3	1	4	4	3
N6	6	4	4	5

Rows show first infection and columns later infections.

No specific pattern on successive infections was found for NA subtypes when performing a full exploration (whole dataset, short and long lag) of the NA subtypes in detected infections, first restricting the analysis to the 2 most common subtypes and then subsequently including the less common subtypes in the contingency tables as done for HA (Tables S15, S16, S17, S18, S19, S20).

## Discussion

### Homosubtypic and heterosubtypic immunity

Here, we have examined the influence of immunity on the likelihood of re-infections of homologous or heterologous HA and NA subtypes. Individual immunity was commonly detected, whereby we detected fewer re-infections with a particular subtype than expected if re-infection was random with respect to viral subtype. For re-infections with homologous subtypes, we found evidence for homosubtypic immunity for the HA protein, but not for the NA protein. This homosubtypic HA immunity was time dependent, whereby it was not observed in cases where the time between isolated viruses was short (1–6 days), but was evident when there were at least 7 days between isolations when immune responses develop. Clearly, the time between detected infections is a crucial parameter for establishing the likelihood that an isolated virus is a new infection, or continued shedding of an already established infection. Isolation of different subtypes from the same bird within a period of less than 3 days likely represents a major fraction of co-infections, rather than separate consecutive infections. Similarly, changes in either HA or NA subtype between viruses isolated within a short period of time from the same bird may signify a reassortment event.

Apart from homosubtypic immunity, we also found evidence for heterosubtypic HA immunity in the wild population of Mallards. The deficit of particular re-infections was evident when analyzing the signs and values of the residuals from pair-wise comparisons in contingency tables. With this approach, phylogenetically related HA subtypes were less likely to be detected than expected, indicating patterns of heterosubtypic immunity in the wild host reservoir. For example, re-infections with the common HA subtypes within the H1 Clade (H1, H2, H5 and H6) were rare if the first detected subtype was an H5 virus. The patterns of HA heterosubtypic immunity were most evident between subtypes belonging to the same clade, but were still present in comparisons between Group 1 (H1, H11 and H9 Clades) and Group 2 (H3 and H7 Clades) viruses. The probability of re-infection of a virus within the same HA clade showed distinct temporal patterns, and decreased below the probability estimated for a naïve individual within ∼7 days. These patterns indicate that once an individual has experienced an infection with a virus from a specific clade, the probability of getting re-infected with a virus from the same clade decrease over time. Individuals develop an immune response, homosubtypic and heterosubtypic immunity, against viruses from that clade and the detection probability remained lower than for naïve individuals up to a month. In contrast, the detection probability of a virus from a specific clade for individuals that experienced a previous infection with a virus from another clade increased above the estimated probability for naïve individuals, as immune responses developed. Consequently these individuals are more likely to be infected by a virus belonging to a different clade than with a subtype from the same clade as the previous infection. Despite low prevalence rates of H11, H7 and H9 Clades, the modeled probabilities of re-infection showed similar shapes to those of the most common subtypes.

Patterns of homosubtypic immunity have been observed in experimental infection studies in both domestic and wild ducks [10], [12]. The duration of protection reported from such studies seems to be comparable to the time span that was observed in this study (at least 30 days). In fact, we only found two clear cases of homosubtypic re-infections, occurring 47 and 49 days, respectively, after the first detected infections. HI and neutralizing antibodies have been detected after experimental infections, from 16 days post infection and lasting only a few weeks [26]. In contrast, the evidence for heterosubtypic immunity from experimental infection studies is more variable. In a recent study, Mallards challenged with a LPAI H5N2 virus 35 days after an initial challenge with LPAI H7N7 virus showed fewer infections and less/lower shedding compared to ducks that had not been pre-challenged [12]. Similarly, Costa and colleagues have shown a reduction in both the duration of shedding and the viral loads in heterosubtypic re-infections (H5N2×H3N8 and H3N8×H5N2) compared to primary infections [11]. Other studies have shown that pre-challenge with heterosubtypic LPAI viruses can give some protection against an otherwise lethal H5N1 HPAI virus infection [9], [17]–[19], [34]. These experimental studies provide valuable insights into the role of homo- and heterosubtypic immunity in IAV infections, especially for circulation of HPAI H5N1, but are limited to few birds and virus subtypes. Our study is based on natural infections from a large sample of wild birds, in an environment where many IAVs subtypes co-circulate and thus presents the first evidence of heterosubtypic immunity in a complex natural system.

In our study population, heterosubtypic immunity seems to develop during the comparatively short period (average 3 weeks) that the ducks stay in the study area. Even though trapping was conducted every day, the infection histories of the birds were for most cases incomplete at a daily level. Furthermore, although isolation rates from samples collected at our site are high [35], still only 44% of all PCR-positive samples yielded a virus isolate, which together precluded detailed analyses of exact shedding times for individual infections. If prior infection is related to a decrease in the duration and intensity of future infections, those future infections will be less likely to be detected. Given the generally high prevalence at our site and short shedding times [31], [32], re-infections and co-infections [36], [37] are certainly common in the population. The prediction from our population-based investigation is that the number of potential co-infections will diminish as the season progresses, due to a build-up of immunity to infecting subtypes (both homo- and hetero-subtypic). Thus, the frequency of reassortment will also be affected by the cross-protective immunity landscape in individual hosts, and will vary during the season at the population level. Sampling of duck populations on European wintering grounds show high seroprevalence of antibodies, and also cases of seroconversion in recaptured ducks [38], [39], but whether individuals develop long-term immunity to IAV is mostly unknown, as repeated sampling over time is rare. The temporal model for heterosubtypic immunity at the Ottenby stopover site indicated a response lasting at least 30 days, which is similar to the average time that birds stay in the area. Our results agree with seroprevalence estimates from wintering grounds and experimental studies following the dynamics of antibodies [15] in suggesting that heterosubtypic immunity apparently last longer and may be boosted by secondary IAV contacts in natural systems.

The specific mechanism conferring protection to IAV, and the respective roles of innate, humoral and cell-mediated immune responses in Mallards, is poorly understood. Heterosubtypic immunity in mice and humans is determined by cross-reactive antibodies (against the HA stem region, NA, M2 and NP) and T cells (CD4+ T helper cells and CD8+ cytotoxic cells) [4]. Furthermore, specific cell-mediated responses in the mucosal tissues, the bronchus-associated lymphoid tissue, and in the gut-associated lymphoid tissue can modulate the outcome of infections [20]. In poultry, activity of cross-reactive CD8+ cytotoxic T cells induced by a H9N2 infection have been shown to cause protection to later HPAI H5N1 challenges [34], [40]. Some studies have reported highly conserved inter-subtype immunodominant epitopes that could explain cross-reactivity [41]. Similarly, the “broadly neutralizing antibodies” observed in humans and mice confer cross-reactive activity and neutralize multiple IAVs subtypes from Group 1 and 2 [42], [43]. Nevertheless, individual variation in immune responses can be expected, determined by genetic predisposition of the host, previous infection history, condition of the host and interactions with the environment.

### Evolution of IAV: How can subtype diversity be maintained locally and globally?

Circulation of multiple subtypes can be sustained when the host population is large enough and will depend on the duration, strength and extent of cross-protective immunity. The Mallard is one of the key reservoir species for IAV [2] and is the most abundant and widespread duck species in the Northern Hemisphere, with an estimated population of 4.5 million individuals in Northwestern Europe [44]. Homo- and heterosubtypic immunity in the Mallard is therefore likely to affect viral dynamics in this system and affect year-round virus circulation in populations with herd immunity lasting during winter and spring.

Some HA subtypes in birds seem to have evolved in an allopatric context and show clear patterns of either geographic isolation, for example the H15 subtype is only found in Australia [45], or host isolation, in the case of H13 and H16 that seem largely restricted to gulls and terns [6], [46]. All other known avian HA subtypes today occur in sympatry in most of the virus' geographic range, even if there is a clear distinction between Eurasian and American lineages. Whether distinct HA subtypes evolved in allopatry and now occur in sympatry, or whether delineation may be selected in a sympatric host population, is unknown. These models are not exclusive and HA subtypes have originated at different times, showing higher-order clustering [7]. Some subtypes, such as H13 and H16, appear to have diverged much more recently [6], [46]. Interestingly, a recent study identified a novel IAV in bats from Guatemala [47], with genes, including a putative H17 HA variant, that suggest that this virus constitutes a new lineage, different from other known IAVs, clearly supporting allopatric diversification.

Immune pressure in the host population, in combination with the high mutation rate of influenza virus (of the order of 10^−3^ substitutions per site per year [48]), have generated antigenically and phylogenetically distinct HA variants through antagonistic selection. HA subtypes are highly divergent with limited intersubtype genetic similarities. For instance, the average amino acid identity within a HA subtype is estimated at >92%, compared to 45.5% (or 38.5% at the HA1 domain) between HA subtypes [7]. Cross-reactivity in HI tests is sometimes observed at high HA sequence similarities between H2 and H5, H7 and H15, or H4 and H14 [49], indicative of cross-protection between related subtypes as observed in the current study. The NA gene has likely evolved in a similar way to that of HA [7]. However, in the analyses conducted here we did not detect any specific patterns of NA transitions in re-infections. NA antibodies cannot directly neutralize the virus infection, and NA antibodies appear to be less important than those against HA to confer protection, but can limit NA-activity and thereby reduce infection severity [4].

To conclude, we detected both homo- and heterosubtypic HA immunity in naturally infected Mallards. This provides important insights into IAV evolution in particular and the processes of pathogen diversification in general. The degree of cross-immunity will play an important role in determining the direction and strength of selection on individual viral subtypes, and thus their evolutionary trajectories. Future research should target mechanisms of IAV immunity in birds, and predictions on heterosubtypic immunity should be assessed by rigorous infection experiments and further studies conducted in natural populations.

## Materials and Methods

### Ethical statement

All handling of birds was performed by trained ornithologists from Ottenby Bird Observatory. The sampling protocol was approved by Linköping Animal Research Ethics Board (permit numbers 8-06, 34-06, 80-07, 111-11, 112-11).

### Sampling

Wild Mallards were caught in a live-duck trap at Ottenby Bird Observatory, Sweden (56°12′N 16°24′E) during the ice-free period of the year (March/April–December) from fall 2002 until December 2009. The trap was emptied daily and all birds taken inside a mobile field laboratory for ringing, measurements and sampling. Samples for IAV detection were taken either by swabbing the cloacae or collecting fresh faeces at the bottom of single-use cardboard boxes. Samples were preserved in transport media (Hanks balanced salt solution containing 0.5% lactalbuminm, 10% glycerol, 200 U/ml penicillin, 200 µg/ml streptomycin, 100 U/ml polymyxin B sulfate, and 250 µg/ml gentamycin, and 50 U/ml nystatin; Sigma) at −70°C. More details on trapping, sampling and storage can be found in previous publications [32], [50].

### IAV detection, isolation and characterization

Infection status was assessed by different RRT-PCR assays targeting the IAV matrix gene [32], [51]. PCR-positive samples were further tested by H5- and H7-specific RRT-PCRs, followed by sequencing of the amplicon [52] to ensure that all viruses were classified as LPAI before virus propagation. For isolation, specific pathogen-free embryonated hens' eggs were used according to standard methods for IAV propagation [53]. The HA subtype of virus isolates was characterized using a Hemagglutination Inhibition assay (HI), with antisera raised to all 16 determined HA variants. The NA subtype was characterized by PCR and sequencing [35], [54]. The HA gene of some selected isolates was also sequenced. In these cases, a fragment of approximately 600 bp was amplified using the primers HA-1134-F and Bm-NS-890 [55] and sequenced using standard methods. The HA and NA sequences were aligned and explored using BioEdit version 7.0.0 [56].

### Duration of infection and different infections in re-captured Mallards

Following a similar approach to a previous study [31], we estimated the minimal duration of active viral shedding in infected individuals. However, we only used data from typed isolates (HA or HA/NA level depending on whether the NA had been successfully sequenced), and assessed the number of days between first and last isolation of the same virus subtype in an individual. Shedding was intermittent in some individuals and for these we calculated the number of days between first and last isolation of the same virus subtype when there was only one negative sampling day (negative in culture, or negative in the RRT-PCR) between isolations.

The number of days between isolations of a different subtype (HA or HA/NA combination) or re-infection with the same subtype (≥7 days between recovery of the same HA and thus clearly representing different infections) was calculated. In cases of several isolates from the same infection, the number of days was calculated from the last day of detected virus shedding (isolate) until the next subtype. Viruses of the same HA subtype isolated from the same individual within a time period of 3 to 10 days (in addition to two apparent homosubtypic re-infections) were subjected to HA/NA sequencing to determine whether the infection detected was caused by the same virus or by a different virus of the same HA subtype (KC342592-KC342631). Pairs of HAs showed high sequence identity and therefore some cases were not included as transitions in the analyses since the same virus was detected. When analyzing the NA sequences, we also found cases with high sequence similarities, indicating intermittent or long shedding of the same infection. However, for other individuals we detected a different NA subtype between sampling occasions, indicating co-infection events and/or isolation of reassorted viruses.

Virus diversity was high at the study site and during the complete study period (2002–2009) 12 different HA subtypes were detected: H4 (*n* isolates = 291), H1 (*n* isolates = 141), H11 (*n* isolates = 118), H6 (*n* isolates = 105), H5 (*n* isolates = 99), H2 (*n* isolates = 96), H3 (*n* isolates = 74), H10 (*n* isolates = 63), H7 (*n* isolates = 41), H8 (*n* isolates = 19), H12 (*n* isolates = 14), H9 (*n* isolates = 9). The 7 most frequent subtypes were recorded every year, while the others occurred intermittently and more rarely. In the analyses, subtypes were grouped in HA clades: H1 Clade (H1, H2, H5, H6), H3 Clade (H3, H4), H11 Clade (H11), H7 Clade (H7, H10), H8 Clade (H8, H9, H12); and HA groups: Group 1 (H1 Clade, H9 Clade, and H11 Clade) and Group 2 (H3 Clade and H7 Clade). All NA subtypes (N1 to N9) were detected during the study period, most subtypes were detected every year except for N5 and N7 that were absent some years. NA subtypes could be ordered in frequency: N6 (*n* isolates = 231), N2 (*n* isolates = 217), N1 (*n* isolates = 125), N3 (*n* isolates = 116), N9 (*n* isolates = 80), N8 (*n* isolates = 54), N7 (*n* isolates = 41), N5 (*n* isolates = 35), N4 (*n* isolates = 26). NA subtypes were grouped into clades and groups in the analyses: N3 Clade (N2, N3), N7 Clade (N6, N7, N9), N4 Clade (N1, N4), N8 Clade (N5, N8); NA Group 1 (N4 Clade and N8 Clade) and Group 2 (N3 Clade and N7 Clade).

### Epidemiological and statistical analysis

All statistical tests and models were computed using the R software [57].

#### Minimum duration of shedding

Minimum duration of shedding was modelled and estimated using generalized linear models with the number of days between first and last isolation of a same virus as the response variable, and month, year and HA subtype as categorical explanatory variables. As the response variable was a count, the models were set up using a Poisson distribution and a log link function. Statistical significance of the explanatory variables was assessed using likelihood ratio tests (LRT). Estimations of minimum shedding duration were derived from the model including only statistically significant explanatory variables.

#### Dependency between consecutive infections

We constructed contingency tables with the HA or NA subtypes from consecutive infections (rows for first infection and columns for later infection), including consecutive isolation of different viruses or isolations between negative samples (thus detections defined as belonging to the same infection were not included). The consecutive infections contingency tables were sparse with frequencies being null in most cells and rarely exceeding five. This implied that asymptotic approaches such as standard chi squared tests could not be applied. Instead, for testing independence null hypotheses we relied on exact inference methods for categorical data [58], [59] using the Fisher's exact test implemented in the “fisher.test” function of R. For contingency tables of dimensions less than 6*6, the *p*-value for the null hypothesis was determined using a network algorithm developed by Mehta and Patel [60] that allows the full enumeration of the contingency tables with margins similar to those of the observed table and the computation of the probability of each of these tables under the null hypothesis of independence. With this approach, the exact *p*-value of the observed contingency table (referred to as Fisher's exact test in the result section) is computed as the sum of the hypergeometric probabilities under the null hypothesis of independence of the tables as likely as, or less likely than the observed table. For contingency tables of dimensions greater than 6*6, this algorithm was computationally intractable. The *p*-value for the null hypothesis was then determined using a Monte Carlo approach (option “simulate.p.value = TRUE” in the R fisher.test function), where 10 000 contingency tables are sampled in proportion to their hypergeometric probabilities from the set of tables with margins similar to those of the observed table. The *p*-value of the observed table (referred to as MC Fisher's test in the result section) is then computed as the proportion of the sampled tables which hypergeometric probability is lower or equal to that of the observed contingency table [58], [59]

Moreover, because 20 individuals had more than two detected infections, they contributed with more than one pair of infection events to some contingency tables. In order not to violate the test statistic assumption of independent statistical units, but still making full use of the available data, we adhered to a re-sampling strategy, based on 1000 contingency tables built using random subsamples including only one pair of infection events per individual (based on unique ring numbers of birds). The median *p*-value over these subsamples was used for assessing the probability of incorrectly rejecting the independence null hypothesis. The specific patterns of departure from independence in the tables that included all pairs of infections (even multiple pairs of infections from single individuals), was examined using standardized Pearson's residuals *r_ij_*. For the cell at intersection of line i and column j:
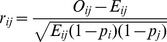
where *O_ij_* is the observed cell frequency, *E_ij_* is the expected cell frequency under the independence hypothesis, *p_i_* is the marginal relative frequency for row *i* and *p_j_* is the marginal relative frequency for column *j*. Positive residuals reflect an excess of observed over expected cases under the assumption that t subtype of the second infection is independent from the subtype of the first infection. Conversely, negative residuals reflected a deficit of observed cases as compared to the expected cases.

We fully explored a range of contingency tables starting with the 2 or 3 most common subtypes and subsequently including less frequent subtypes one by one. We computed the mean of the standardized Pearson residuals for different categories of cells and distinguished cells corresponding to infections by the same subtype, cells corresponding to infections by different subtypes belonging to the same clade and cells corresponding to infections by subtypes belonging to different clades. Similar tests were done for the HA clade and group levels, and for NA subtypes.

#### Patterns of infection detection probabilities over time

We built statistical models to address variation in the probability of a virus belonging to a given HA clade, conditional on detection and identification. For these analyses, all records of infections for which the virus subtype was known were considered, thus including all records of detections likely to belong to the same infection. One distinct analysis was performed for each clade (H1, H3, H11, H7 and H9 Clades). We used the mgcv package in R for fitting generalized additive models (GAMs) with the possibility of including non-parametric smoothers [61]. The dependent variable was binary: membership of the subtype to the focal clade or membership to another clade, and thus had a binomial distribution (with number of trials equaling one) and a logit link function. This model was used to depict the relationship between the probability parameter of the binomial distribution and explanatory variables. The explanatory variables considered were: (1) year (categorical), (2) whether or not a virus belonging to the same clade had previously been detected in the same individual (binary), and (3) whether or not a virus belonging to a different clade had previously been detected in the same individual (binary). Additionally, non-parametric smooth functions were included to address the influence of (1) date (seasonal variation) (2) the number of days since the last detection in the same individual of a virus belonging to the same clade, and (3) the number of days since the last detection in the same individual of a virus belonging to a different clade. The GAMs were fitted using penalized likelihood maximization. The smooth terms were thin plate regression splines with smoothing parameters estimated by minimization of the generalized cross validation criterion. For each clade, a set of models including distinct combinations of the explanatory variables and smooth functions described above were fitted to the data. No interaction between parameters was included in these models, in order to avoid over-parametrization. Selection within each such set of the most appropriate model(s) was performed using the Akaike Information Criterion (AIC), whereby AIC models with the lowest scores were considered to provide the best description of the data.

## Supporting Information

Figure S1 **Distribution of days between different detected IAV infections.**
(TIF)

Figure S2 **Distribution of** ***p*** **-values over randomly generated independent subsamples for the long lag (≥7 days between isolation) and common subtypes.** (A) *p*-value distribution of MC Fisher's test at the level of subtype; (B) *p*-value distribution of Fisher's test at the level of clade; (C) *p*-value distribution of MC Fisher's test at the level of group.
(TIF)

Figure S3 **Prevalence estimate of H1 Clade and H3 Clade viruses as a function of time.** The line gives a daily prevalence estimate for H1 Clade (A) and H3 Clade (B) viruses (with 95% confidence limits in dashed lines) calculated from the total study period 2002–2008. The distribution of data points is presented as rug plots along the x-axis.
(TIF)

Figure S4 **IAV detection probability for H7, H9 and H11 Clades as a function of time and previously detected infections.** The x-axis represents time in days since first detection of a virus. The y-axis depicts the probability of detection. Black continuous line represents the change over time in probability (95% CI with dashed lines). The horizontal blue line is the probability of detection of an infection with a virus from a specific clade for naïve birds (95% CI with dashed lines). The distribution of data points is presented as rug plots along the x-axis. Detection probabilities for H7 (A), H9 (C) and H11 (E) Clades for individuals that have previously experienced an initial infection with a virus from the same clade. Detection probabilities for H7 (B), H9 (D) and H11 (F) Clades for individuals that have previously experienced an infection with a virus from another clade.
(TIF)

Figure S5 **Prevalence estimate of H7 Clade, H9 Clade and H11 Clade viruses as a function of time.** The line gives a daily prevalence estimate for H7 Clade (A), H9 Clade (B) and (C) H11 Clade viruses (with 95% confidence limits in hatched lines) calculated from the total study period 2002–2008. The distribution of data points is presented as rug plots along the x-axis.
(TIF)

Table S1 **Contingency table for all HA re-infection transitions, rows first infection, columns later infection.**
(DOCX)

Table S2 **Contingency table for phylogenetic HA group independence.**
(DOCX)

Table S3 **Contingency table for phylogenetic HA clade independence.**
(DOCX)

Table S4 **Contingency table for HA re-infections (short lag).**
(DOCX)

Table S5 **Contingency table for phylogenetic HA clade independence (short lag).**
(DOCX)

Table S6 **Contingency table for phylogenetic HA group independence (short lag).**
(DOCX)

Table S7 **Contingency table for phylogenetic HA group independence (long lag, all subtypes).**
(DOCX)

Table S8 **Summary table of the exploration of contingency tables at the HA subtype level for the long lag.**
(DOC)

Table S9 **Summary table of the exploration of the contingency tables at the HA clade level for the long lag.**
(DOC)

Table S10 **Summary table of the exploration of contingency tables at the HA subtype level for the whole dataset.**
(DOC)

Table S11 **Summary table of the exploration of contingency tables at the HA subtype level for the short lag.**
(DOC)

Table S12 **Summary table of the exploration of the contingency tables at the HA clade level for the whole dataset.**
(DOC)

Table S13 **Summary table of the exploration of the contingency tables at the HA clade level for the short lag.**
(DOC)

Table S14 **Alternative models for depicting the probability that a detected and identified virus belong to a focal clade.** Model number one includes all the candidate explanatory variables. In each of models 2–7 one or two explanatory variables are removed from model number one. The AICs of selected models are highlighted in bold.
(DOC)

Table S15 **Summary table of the exploration of the contingency tables at the NA subtype level for the whole dataset.**
(DOC)

Table S16 **Summary table of the exploration of the contingency tables at the NA subtype level for the short lag.**
(DOC)

Table S17 **Summary table of the exploration of the contingency tables at the NA subtype level for the long lag.**
(DOC)

Table S18 **Summary table of the exploration of the contingency tables at the NA clade level for the whole dataset.**
(DOC)

Table S19 **Summary table of the exploration of the contingency tables at the NA clade level for the short lag.**
(DOC)

Table S20 **Summary table of the exploration of the contingency tables at the NA clade level for the long lag.**
(DOC)
