# Supplementary figures and images for: Heterosubtypic Immunity to Influenza A Virus Infections in Mallards May Explain Existence of Multiple Virus Subtypes
Source: PLoS Pathog. 2013 Jun 20;9(6):e1003443. doi: 10.1371/journal.ppat.1003443 (PMC3688562; doi:10.1371/journal.ppat.1003443)

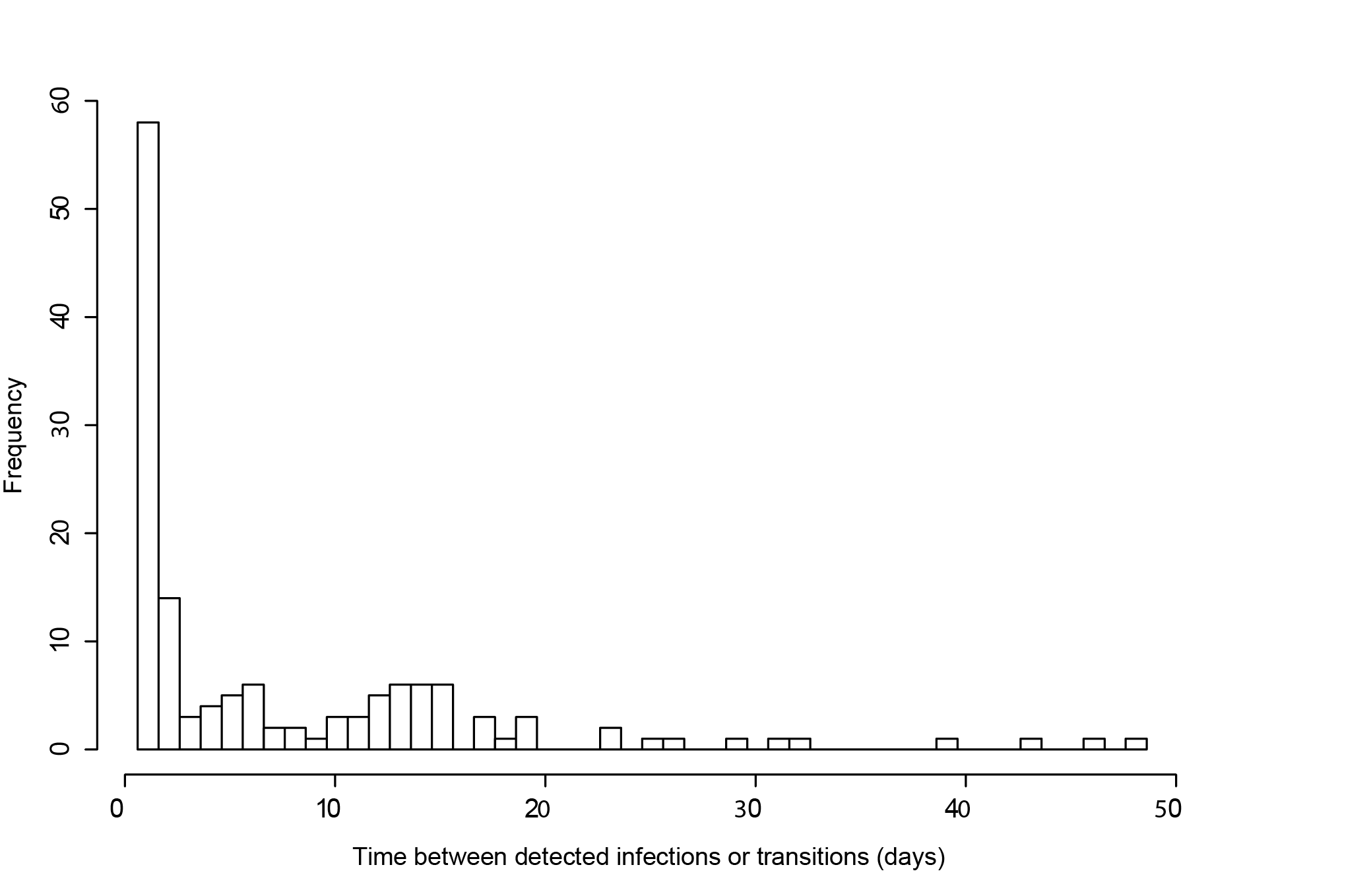

Supplement: Figure S1 — Distribution of days between different detected IAV infections. (TIF) [file ppat.1003443.s001.tif]

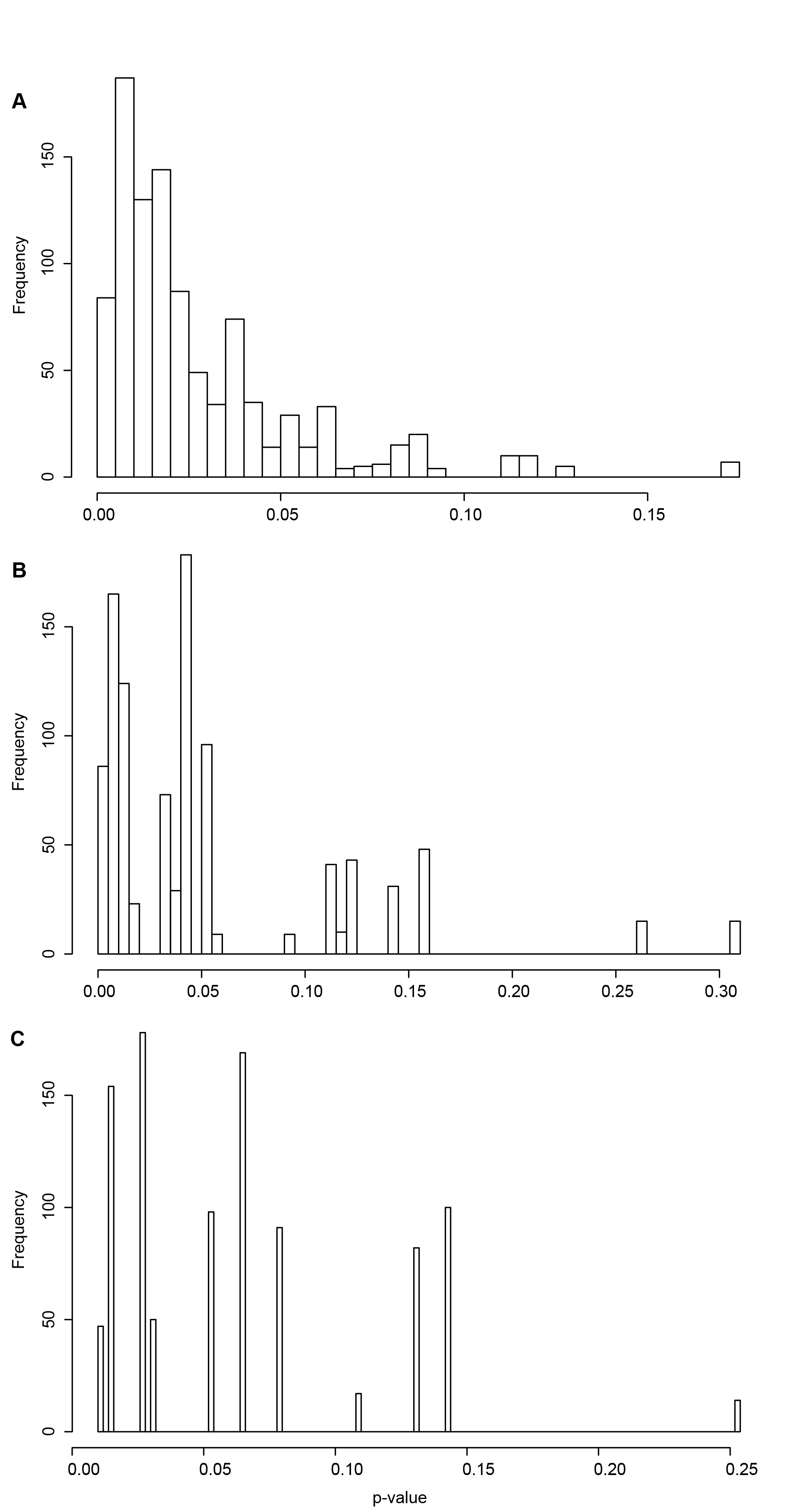

Supplement: Figure S2 — Distribution of p -values over randomly generated independent subsamples for the long lag (≥7 days between isolation) and common subtypes. (A) p-value distribution of MC Fisher's test at the level of subtype; (B) p-value distribution of Fisher's test at the level of clade; (C) p-value distribution of MC Fisher's test at the level of group. (TIF) [file ppat.1003443.s002.tif]

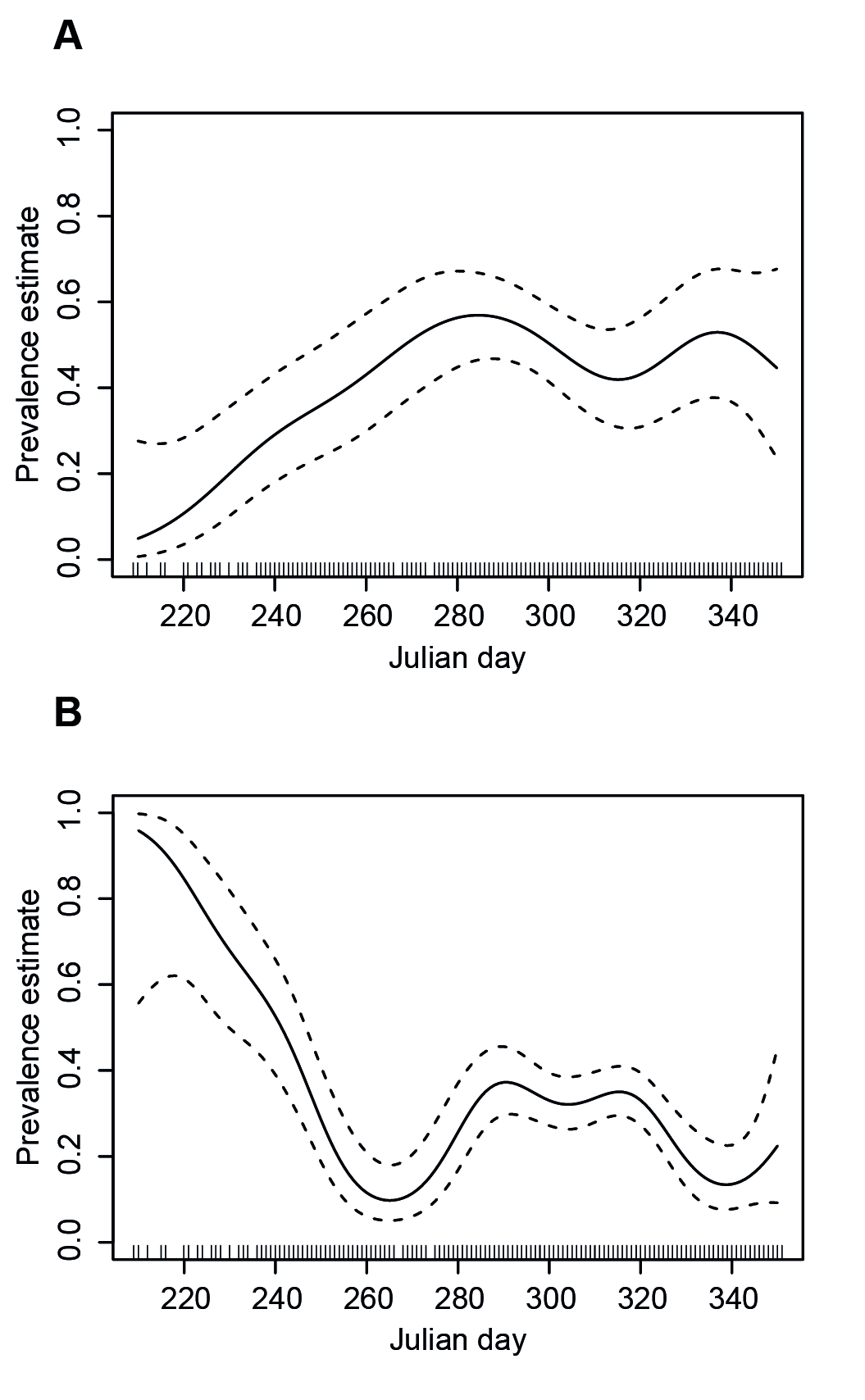

Supplement: Figure S3 — Prevalence estimate of H1 Clade and H3 Clade viruses as a function of time. The line gives a daily prevalence estimate for H1 Clade (A) and H3 Clade (B) viruses (with 95% confidence limits in dashed lines) calculated from the total study period 2002–2008. The distribution of data points is presented as rug plots along the x-axis. (TIF) [file ppat.1003443.s003.tif]

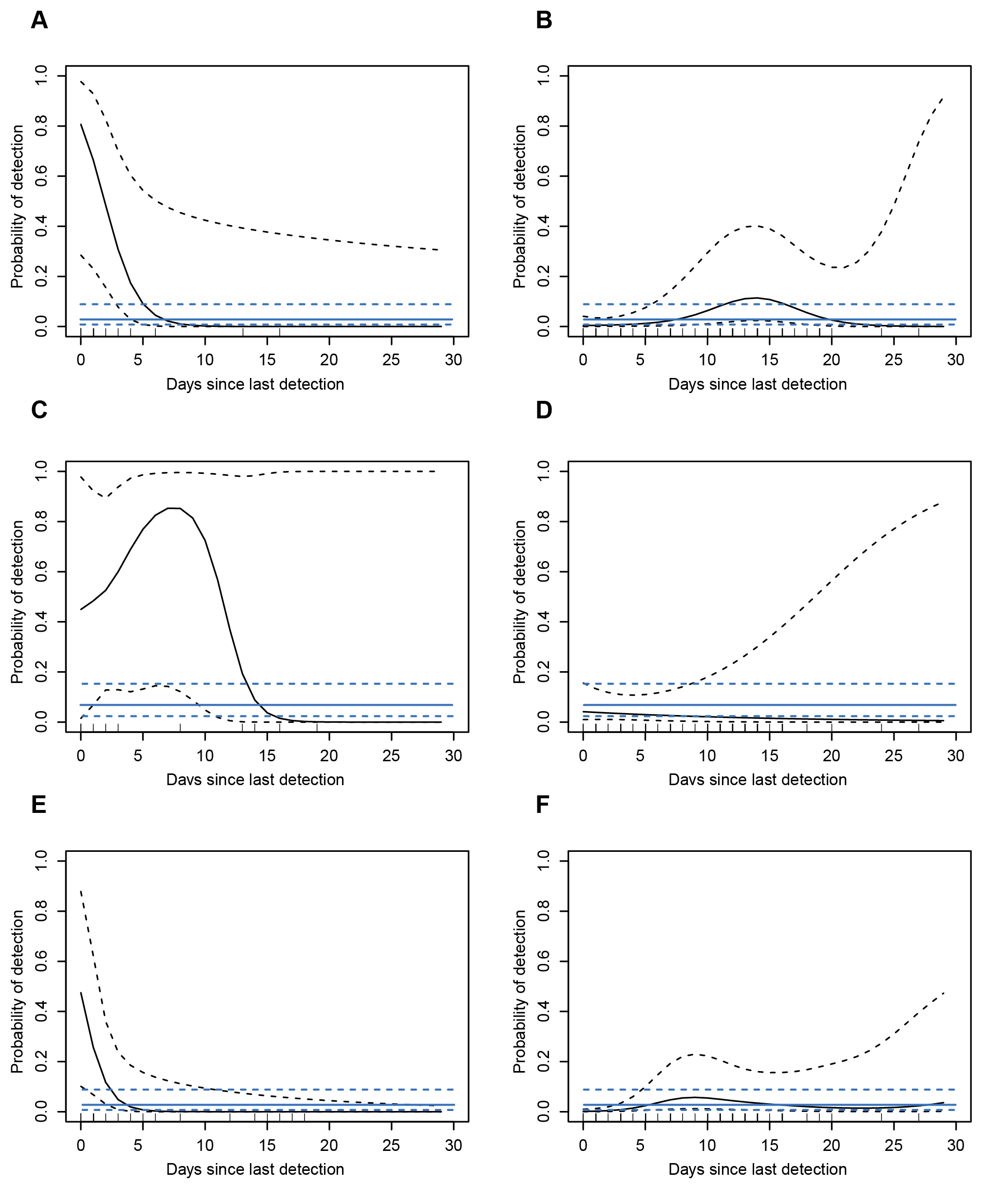

Supplement: Figure S4 — IAV detection probability for H7, H9 and H11 Clades as a function of time and previously detected infections. The x-axis represents time in days since first detection of a virus. The y-axis depicts the probability of detection. Black continuous line represents the change over time in probability (95% CI with dashed lines). The horizontal blue line is the probability of detection of an infection with a virus from a specific clade for naïve birds (95% CI with dashed lines). The distribution of data points is presented as rug plots along the x-axis. Detection probabilities for H7 (A), H9 (C) and H11 (E) Clades for individuals that have previously experienced an initial infection with a virus from the same clade. Detection probabilities for H7 (B), H9 (D) and H11 (F) Clades for individuals that have previously experienced an infection with a virus from another clade. (TIF) [file ppat.1003443.s004.tif]

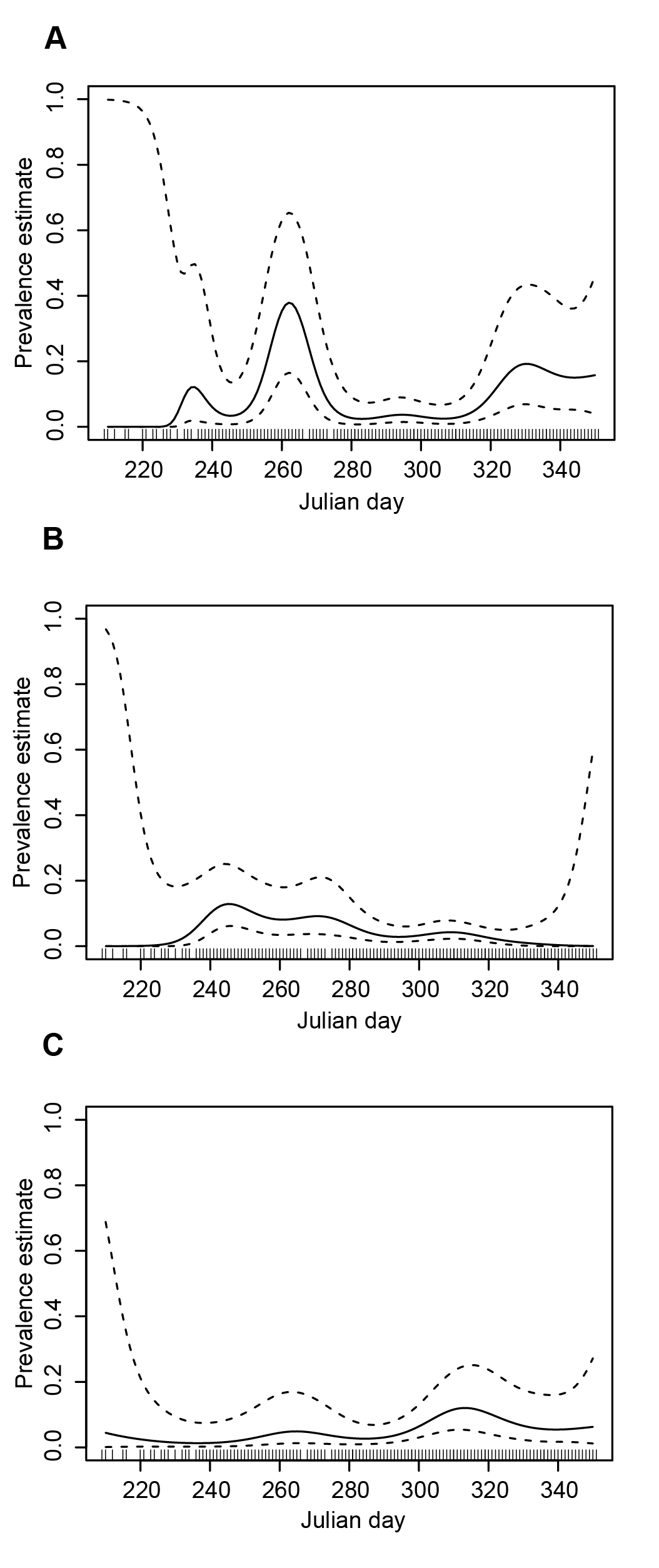

Supplement: Figure S5 — Prevalence estimate of H7 Clade, H9 Clade and H11 Clade viruses as a function of time. The line gives a daily prevalence estimate for H7 Clade (A), H9 Clade (B) and (C) H11 Clade viruses (with 95% confidence limits in hatched lines) calculated from the total study period 2002–2008. The distribution of data points is presented as rug plots along the x-axis. (TIF) [file ppat.1003443.s005.tif]
