# Supplementary material for: Heterosubtypic Immunity to Influenza A Virus Infections in Mallards May Explain Existence of Multiple Virus Subtypes
Source: PLoS Pathog. 2013 Jun 20;9(6):e1003443. doi: 10.1371/journal.ppat.1003443 (PMC3688562; doi:10.1371/journal.ppat.1003443)
Supplement: Table S2 — Contingency table for phylogenetic HA group independence. (DOCX) [file ppat.1003443.s007.docx]

**Table S2.** Contingency table for phylogenetic HA Group independence.

|  | 2nd infection | |
| --- | --- | --- |
| 1st infection | Group 1 | Group 2 |
| Group 1 | 36 | 42 |
| Group 2 | 40 | 24 |
