# Supplementary material for: Heterosubtypic Immunity to Influenza A Virus Infections in Mallards May Explain Existence of Multiple Virus Subtypes
Source: PLoS Pathog. 2013 Jun 20;9(6):e1003443. doi: 10.1371/journal.ppat.1003443 (PMC3688562; doi:10.1371/journal.ppat.1003443)
Supplement: Table S5 — Contingency table for phylogenetic HA clade independence (short lag). (DOCX) [file ppat.1003443.s010.docx]

**Table S5**. Contingency table for phylogenetic HA Clade independence (short lag).

|  | 2^nd^ infection | | | | |
| --- | --- | --- | --- | --- | --- |
| 1^st^ infection | H1 Clade | H3 Clade | H7 Clade | H9 Clade | H11 Clade |
| H1 Clade | 12 | 14 | 2 | 1 | 1 |
| H3 Clade | 14 | 12 | 1 | 1 | 5 |
| H7 Clade | 2 | 2 | 2 | 2 | 0 |
| H9 Clade | 1 | 1 | 0 | 0 | 0 |
| H11 Clade | 3 | 5 | 0 | 1 | 2 |
