# Supplementary material for: Heterosubtypic Immunity to Influenza A Virus Infections in Mallards May Explain Existence of Multiple Virus Subtypes
Source: PLoS Pathog. 2013 Jun 20;9(6):e1003443. doi: 10.1371/journal.ppat.1003443 (PMC3688562; doi:10.1371/journal.ppat.1003443)
Supplement: Table S6 — Contingency table for phylogenetic HA group independence (short lag). (DOCX) [file ppat.1003443.s011.docx]

**Table S6.** Contingency table for phylogenetic HA group independence (short lag).

|  | 2^nd^ infection | |
| --- | --- | --- |
| 1^st^ infection | Group 1 | Group 2 |
| Group 1 | 21 | 22 |
| Group 2 | 24 | 17 |
