# Supplementary material for: Heterosubtypic Immunity to Influenza A Virus Infections in Mallards May Explain Existence of Multiple Virus Subtypes
Source: PLoS Pathog. 2013 Jun 20;9(6):e1003443. doi: 10.1371/journal.ppat.1003443 (PMC3688562; doi:10.1371/journal.ppat.1003443)
Supplement: Table S7 — Contingency table for phylogenetic HA group independence (long lag, all subtypes). (DOCX) [file ppat.1003443.s012.docx]

**Table S7**. Contingency table for phylogenetic HA Group independence (long lag, all subtypes).

|  | 2^nd^ infection | | | | | | | | | | | |
| --- | --- | --- | --- | --- | --- | --- | --- | --- | --- | --- | --- | --- |
| 1^st^ infection | H1 | H2 | H3 | H4 | H5 | H6 | H7 | H8 | H9 | H10 | H11 | H12 |
| H1 | 0 | 0 | 2 | 1 | 0 | 3 | 0 | 0 | 0 | 2 | 0 | 0 |
| H2 | 0 | 0 | 0 | 1 | 0 | 0 | 0 | 0 | 0 | 0 | 0 | 0 |
| H3 | 2 | 0 | 1 | 0 | 0 | 1 | 0 | 0 | 0 | 0 | 0 | 0 |
| H4 | 0 | 3 | 0 | 1 | 2 | 3 | 1 | 0 | 0 | 3 | 2 | 0 |
| H5 | 1 | 0 | 0 | 4 | 0 | 0 | 2 | 0 | 0 | 1 | 4 | 0 |
| H6 | 0 | 1 | 0 | 4 | 0 | 0 | 0 | 0 | 0 | 1 | 0 | 0 |
| H7 | 0 | 0 | 0 | 0 | 0 | 0 | 0 | 0 | 0 | 0 | 1 | 0 |
| H8 | 0 | 0 | 0 | 0 | 0 | 0 | 0 | 0 | 0 | 0 | 1 | 0 |
| H10 | 0 | 2 | 0 | 1 | 0 | 0 | 0 | 0 | 0 | 0 | 0 | 0 |
| H11 | 1 | 2 | 0 | 1 | 0 | 1 | 0 | 0 | 0 | 0 | 0 | 0 |
| H12 | 1 | 0 | 0 | 1 | 0 | 0 | 0 | 0 | 0 | 0 | 0 | 0 |
