# Supplementary material for: Heterosubtypic Immunity to Influenza A Virus Infections in Mallards May Explain Existence of Multiple Virus Subtypes
Source: PLoS Pathog. 2013 Jun 20;9(6):e1003443. doi: 10.1371/journal.ppat.1003443 (PMC3688562; doi:10.1371/journal.ppat.1003443)
Supplement: Table S8 — Summary table of the exploration of contingency tables at the HA subtype level for the long lag. (DOC) [file ppat.1003443.s013.doc]

**Table S8.** Summary table of the exploration of contingency tables at the HA subtype level for the long lag.

| **Number of most common subtypes considered** | **3** | **4** | **5** | **6** | **7** | **8** | **9** | **10** | **11** |
| --- | --- | --- | --- | --- | --- | --- | --- | --- | --- |
| Number of cells | 9 | 16 | 25 | 36 | 49 | 64 | 81 | 90 | 99 |
| Number of cells with expected frequency <5 | 9 | 16 | 25 | 36 | 49 | 64 | 81 | 90 | 99 |
| Number of individuals | 6 | 14 | 23 | 29 | 32 | 40 | 42 | 42 | 44 |
| Number of transitions | 6 | 17 | 28 | 35 | 41 | 51 | 55 | 56 | 58 |
| Test for H0: independence on the full table | 0.80 | 0.08 | **0.02** | **0.02** | **0.003*** | **0.01*** | **0.02*** | **0.02*** | **0.02*** |
| Median p-value over 1000 subsamples with a single transition per individual |  | 0.10 | **0.03** | 0.06 | **0.02** | **0.04*** | **0.05*** | **0.05*** | 0.06* |
| Mean Pearson residuals for same subtype cells | -0.84 | -1.18 | -1.13 | -1.04 | -0.64 | -0.61 | -0.55 | 0.54* | -0.55 |
| Mean Pearson residuals for different subtype same clade cells |  | 0.50 | -0.14 | -0.26 | -0.50 | -0.42 | -0.5 | -0.49 | -0.49 |
| Mean Pearson residuals for different clade cells | 0.42 | 0.38 | 0.45 | 0.42 | 0.40 | 0.28 | 0.21 | 0.18 | 0.15 |

* Fisher’s exact p-value for each contingency table computed using a Monte Carlo procedure. Bold p-values indicate significant tables. Restricting the data to the 2 most common subtypes was not possible, as this generated a 2*1 table. The table for all subtypes included 11 subtypes as there were no infections with H9.
