# Supplementary material for: Heterosubtypic Immunity to Influenza A Virus Infections in Mallards May Explain Existence of Multiple Virus Subtypes
Source: PLoS Pathog. 2013 Jun 20;9(6):e1003443. doi: 10.1371/journal.ppat.1003443 (PMC3688562; doi:10.1371/journal.ppat.1003443)
Supplement: Table S9 — Summary table of the exploration of the contingency tables at the HA clade level for the long lag. (DOC) [file ppat.1003443.s014.doc]

**Table S9**. Summary table of the exploration of the contingency tables at the HA clade level for the long lag.

| **Number of most common clades considered** | **2 most common clades** | **3 most common clades** | **4 most common clades** | **All clades** | **Group level- 3 most common clades** | **Group level-All clades** |
| --- | --- | --- | --- | --- | --- | --- |
| Number of cells | 4 | 9 | 16 | 20 | 4 | 4 |
| Number of cells with expected frequency <5 | 0 | 5 | 12 | 16 | 0 | 0 |
| Number of individuals | 23 | 32 | 42 | 44 | 32 | 44 |
| Number of transitions | 30 | 41 | 55 | 58 | 41 | 58 |
| Test for H0: independence on the full table | **0.004** | **0.015** | 0.06 | 0.09 | **0.02** | 0.06 |
| Median p-value over 1000 subsamples with a single transition per individual | **0.009** | **0.04** | 0.18 | 0.28 | **0.05** | 0.07 |
| Mean Pearson residuals for same clade cells | -3.00 | -2.20 | -1.82 | -1.23 | -2.35 | -1.99 |
| Mean Pearson residuals for different clade cells | 3.00 | 1.10 | 0.58 | 0.28 | 2.35 | 1.99 |

* Fisher’s exact p-value for each contingency table computed using a Monte Carlo procedure. Bold p-values indicate significant tables. HA clades are in decreasing frequency order: H1 Clade (H1, H2, H5, H6), H3 Clade (H3, H4), H11 Clade (H11), H7 Clade (H7, H10), H8 Clade (H8, H9, H12). The two HA groups are: Group 1 (H1 Clade, H9 Clade and H11 Clade) and Group 2 (H3 Clade and H7 Clade).
