# Supplementary material for: Heterosubtypic Immunity to Influenza A Virus Infections in Mallards May Explain Existence of Multiple Virus Subtypes
Source: PLoS Pathog. 2013 Jun 20;9(6):e1003443. doi: 10.1371/journal.ppat.1003443 (PMC3688562; doi:10.1371/journal.ppat.1003443)
Supplement: Table S10 — Summary table of the exploration of contingency tables at the HA subtype level for the whole dataset. (DOC) [file ppat.1003443.s015.doc]

**Table S10.** Summary table of the exploration of contingency tables at the HA subtype level for the whole dataset.

| **Number of most common subtypes considered** | **2** | **3** | **4** | **5** | **6** | **7** | **8** | **9** | **10** | **11** | **All** |
| --- | --- | --- | --- | --- | --- | --- | --- | --- | --- | --- | --- |
| Number of cells | 4 | 9 | 16 | 25 | 36 | 49 | 64 | 81 | 100 | 121 | 132 |
| Number of cells with expected frequency <5 | 2 | 6 | 13 | 22 | 34 | 46 | 62 | 79 | 98 | 119 | 130 |
| Number of individuals | 19 | 34 | 46 | 60 | 71 | 83 | 93 | 96 | 98 | 103 | 104 |
| Number of transitions | 19 | 36 | 54 | 73 | 87 | 109 | 127 | 132 | 136 | 141 | 142 |
| Test for H0: independence on the full table | 0.18 | 0.46 | 0.43 | 0.39* | 0.50* | 0.40* | 0.34* | 0.26* | 0.13* | 0.23* | 0.23* |
| Median p-value over 1000 subsamples with a single transition per individual |  | 0.40 | 0.56 | 0.67 | 0.73* | 0.70* | 0.67* | 0.51* | 0.41* | 0.59* | 0.58* |
| Mean Pearson residuals for same subtype cells | -1.53 | -1.09 | -0.74 | -0.81 | -0.59 | -0.55 | 0.11 | 0.11 | -0.03 | -0.04 | -0.03 |
| Mean Pearson residuals for different subtype same clade cells |  |  | 0.04 | -0.13 | -0.13 | -0.28 | -0.33 | -0.33 | 0.30 | -0.29 | -0.28 |
| Mean Pearson residuals for different clade cells | 1.53 | 0.54 | 0.27 | 0.33 | 0.33 | 0.26 | 0.09 | 0.09 | 0.1 | 0.08 | 0.07 |

* Fisher’s exact p-value for each contingency table computed using a Monte Carlo procedure.
