# Supplementary material for: Heterosubtypic Immunity to Influenza A Virus Infections in Mallards May Explain Existence of Multiple Virus Subtypes
Source: PLoS Pathog. 2013 Jun 20;9(6):e1003443. doi: 10.1371/journal.ppat.1003443 (PMC3688562; doi:10.1371/journal.ppat.1003443)
Supplement: Table S11 — Summary table of the exploration of contingency tables at the HA subtype level for the short lag. (DOC) [file ppat.1003443.s016.doc]

**Table S11**. Summary table of the exploration of contingency tables at the HA subtype level for the short lag.

| **Number of most common subtypes considered** | **2** | **3** | **4** | **5** | **6** | **7** | **8** | **9** | **10** | **11** | **All** |
| --- | --- | --- | --- | --- | --- | --- | --- | --- | --- | --- | --- |
| Number of cells | 4 | 9 | 16 | 25 | 36 | 49 | 64 | 81 | 100 | 121 | 132 |
| Number of cells with expected frequency <5 | 2 | 7 | 14 | 23 | 34 | 47 | 62 | 79 | 98 |  | 130 |
| Number of individuals | 17 | 28 | 33 | 40 | 46 | 57 | 62 | 63 | 66 | 69 | 70 |
| Number of transitions | 17 | 30 | 37 | 45 | 52 | 68 | 76 | 77 | 80 | 83 | 84 |
| Test for H0: independence on the full table | 0.29 | 0.53 | 0.46 | 0.47 | 0.39* | 0.44* | 0.39* | 0.27* | 0.13* | 0.17* | 0.17* |
| Median p-value over 1000 subsamples with a single transition per individual |  | 0.52 | 0.40 | 0.53 | 0.45 | 0.49* | 0.39* | 0.27* | 0.13* | 0.18* | 0.19* |
| Mean Pearson residuals for same subtype cells | -1.44 | -0.81 | 0.08 | 0.32 | 0.12 | 0.01 | 0.05 | 0.06 | 0.765 | 0.13 | 0.65 |
| Mean Pearson residuals for different subtype same clade cells |  |  | -0.66 | 0.045 | 0.13 | 0.24 | 0.013 | 0.001 | -0.03 | 0.04 | -0.05 |
| Mean Pearson residuals for different clade cells | 1.44 | 0.40 | 0.10 | -0.03 | -0.09 | 0.003 | 0.014 | -0.10 | -0.05 | -0.04 | -0.03 |

* Fisher’s exact p-value for each contingency table computed using a Monte Carlo procedure.
