# Supplementary material for: Heterosubtypic Immunity to Influenza A Virus Infections in Mallards May Explain Existence of Multiple Virus Subtypes
Source: PLoS Pathog. 2013 Jun 20;9(6):e1003443. doi: 10.1371/journal.ppat.1003443 (PMC3688562; doi:10.1371/journal.ppat.1003443)
Supplement: Table S12 — Summary table of the exploration of the contingency tables at the HA clade level for the whole dataset. (DOC) [file ppat.1003443.s017.doc]

**Table S12.** Summary table of the exploration of the contingency tables at the HA clade level for the whole dataset.

| **Number of most common clades considered** | **2 most common clades** | **3 most common clades** | **4 most common clades** | **All clades** | **Group level 3 most common clades** | **Group level all clades** |
| --- | --- | --- | --- | --- | --- | --- |
| Number of cells | 4 | 9 | 16 | 25 | 4 | 4 |
| Number of cells with expected frequency <5 | 0 | 1 | 6 | 15 | 0 | 0 |
| Number of individuals | 63 | 83 | 96 | 104 | 83 | 104 |
| Number of transitions | 82 | 109 | 132 | 142 | 109 | 142 |
| Test for H0: independence on the full table | **0.03** | 0.22 | 0.39 | 0.34* | **0.05** | 0.06 |
| Median p-value over 1000 subsamples with a single infection event pair per individual | **0.04** | 0.27 | 0.51 | 0.56* | 0.07 | 0.11 |
| Mean Pearson residuals for same clade cells | -2.22 | -1.28 | -0.66 | -0.60 | -2.13 | -1.94 |
| Mean Pearson residuals, for different clade cells | 2.22 | 0.64 | 0.22 | 0.18 | 2.13 | 1.94 |

* Fisher’s exact p-value for each contingency table computed using a Monte Carlo procedure. Bold p-values indicate significant tables. HA clades are in decreasing frequency order: H1 Clade (H1, H2, H5, H6), H3 Clade (H3, H4), H11 Clade (H11), H7 Clade (H7, H10), H8 Clade (H8, H9, H12). The two HA groups are: Group 1 (H1 Clade, H9 Clade and H11 Clade) and Group 2 (H3 Clade and H7 Clade).
