# Supplementary material for: Heterosubtypic Immunity to Influenza A Virus Infections in Mallards May Explain Existence of Multiple Virus Subtypes
Source: PLoS Pathog. 2013 Jun 20;9(6):e1003443. doi: 10.1371/journal.ppat.1003443 (PMC3688562; doi:10.1371/journal.ppat.1003443)
Supplement: Table S13 — Summary table of the exploration of the contingency tables at the HA clade level for the short lag. (DOC) [file ppat.1003443.s018.doc]

**Table S13.** Summary table of the exploration of the contingency tables at the HA clade level for the short lag.

| **Number of most common clades considered** | **2 most common clades** | **3 most common clades** | **4 most common clades** | **All clades** | **Group level 3 most common clades** | **Group level-All clades** |
| --- | --- | --- | --- | --- | --- | --- |
| Number of cells | 4 | 9 | 16 | 20 | 4 | 4 |
| Number of cells with expected frequency <5 | 0 | 5 | 12 | 16 | 0 | 0 |
| Number of individuals | 44 | 57 | 63 | 70 | 57 | 70 |
| Number of transitions | 52 | 68 | 77 | 84 | 68 | 84 |
| Test for H0: independence on the full table | 0.78 | 0.42 | 0.33 | 0.34 | 0.34 | 0.39 |
| Median p-value over 1000 subsamples with a single transition per individual | 0.55 | 0.45 | 0.44 | 0.45 | 0.42 | 0.24 |
| Mean standardized Pearson residuals for same clade cells | -0.55 | 0.03 | 0.80 | 0.55 | -1.04 | -0.89 |
| Mean standardized Pearson residuals, for different clade cells | 0.55 | -0.002 | -0.23 | -0.11 | 1.04 | 0.89 |

* Fisher’s exact p-value for each contingency table computed using a Monte Carlo procedure. HA clades are in decreasing frequency order: H1 Clade (H1, H2, H5, H6), H3 Clade (H3, H4), H11 Clade (H11), H7 Clade (H7, H10), H8 Clade (H8, H9, H12). The two HA groups are: Group 1 (H1 Clade, H9 Clade and H11 Clade) and Group 2 (H3 Clade and H7 Clade).
