# Supplementary material for: Heterosubtypic Immunity to Influenza A Virus Infections in Mallards May Explain Existence of Multiple Virus Subtypes
Source: PLoS Pathog. 2013 Jun 20;9(6):e1003443. doi: 10.1371/journal.ppat.1003443 (PMC3688562; doi:10.1371/journal.ppat.1003443)
Supplement: Table S14 — Alternative models for depicting the probability that a detected and identified virus belong to a focal clade. Model number one includes all the candidate explanatory variables. In each of models 2–7 one or two explanatory variables are removed from model number one. The AICs of selected models are highlighted in bold. (DOC) [file ppat.1003443.s019.doc]

**Table S14.** Alternative models for depicting the probability that a detected and identified virus belong to a focal clade. Model number one includes all the candidate explanatory variables. In each of models 2-7 one or two explanatory variables are removed from model number one. The AICs of selected models are highlighted in bold.

| **Model** | **Parameters** | **H1 Clade** | **H3 Clade** | **H11 Clade** | **H7 Clade** | **H9 Clade** |
| --- | --- | --- | --- | --- | --- | --- |
| 1 | - previous infection by a virus belonging to the same Clade (binary y/n, fixed effect) - number of days elapsed since last detection of a virus belonging to the same Clade (smooth function) - previous infection by a virus belonging to a different Clade (binary y/n, fixed effect) - number of days elapsed since last detection of a virus belonging to a different Clade (smooth function) - year (categorical fixed effect) - date (smooth function) | **1295.32** | 1204.79 | **613.71** | **472.38** | 324.61 |
| 2 | -year | 1307.36 | **1199.72** | 623.74 | 511.69 | **320.97** |
| 3 | - date (smooth function) | 1312.16 | 1255.22 | 624.87 | 509.11 | 334.82 |
| 4 | - previous infection by a virus belonging to the same Clade (binary y/n, fixed effect)  -number of days elapsed since last detection of a virus belonging to the same Clade (smooth function) | 1326.89 | 1262.72 | 638.57 | 517.08 | 334.15 |
| 5 | -number of days elapsed since last detection of a virus belonging to the same Clade (smooth function) | 1320.84 | 1229.94 | 625.80 | 486.59 | 327.15 |
| 6 | -previous infection by a virus belonging to a different Clade (binary y/n, fixed effect)  - number of days elapsed since last detection of a virus belonging to a different Clade (smooth function) | 1341.81 | 1216.54 | 632.48 | 482.73 | 323.85 |
| 7 | - number of days elapsed since last detection of a virus belonging to a different Clade (smooth function) | 1316.83 | 1213.39 | 622.90 | 484.05 | 324.48 |
