# Supplementary material for: Heterosubtypic Immunity to Influenza A Virus Infections in Mallards May Explain Existence of Multiple Virus Subtypes
Source: PLoS Pathog. 2013 Jun 20;9(6):e1003443. doi: 10.1371/journal.ppat.1003443 (PMC3688562; doi:10.1371/journal.ppat.1003443)
Supplement: Table S15 — Summary table of the exploration of the contingency tables at the NA subtype level for the whole dataset. (DOC) [file ppat.1003443.s020.doc]

**Table S15.** Summary table of the exploration of the contingency tables at the NA subtype level for the whole dataset.

| **Number of most common subtypes considered** | **2** | **3** | **4** | **5** | **6** | **7** | **8** | **9** |
| --- | --- | --- | --- | --- | --- | --- | --- | --- |
| Number of cells | 4 | 9 | 16 | 25 | 36 | 42 | 56 | 81 |
| Number of cells with expected frequency <5 | 1 | 4 | 12 | 21 | 33 | 40 | 52 | 78 |
| Number of individuals | 20 | 32 | 48 | 56 | 60 | 63 | 67 | 70 |
| Number of transitions | 21 | 42 | 61 | 71 | 75 | 80 | 87 | 92 |
| Test for H0: independence on the full table | 1.00 | 0.47 | 0.59 | 0.58* | 0.36* | 0.47* | 0.50* | 0.38* |
| Median p-value over 1000 subsamples with a single transition per individual |  | 0.79 | 0.59 | 0.79 | 0.59* | 0.74* | 0.85* | 0.78* |
| Mean Pearson residuals for same subtype cells | -0.13 | -0.81 | -0.12 | 0.12 | 0.69 | 0.69 | 0.91 | 0.69 |
| Mean Pearson residuals for different subtype same clade cells |  |  | -0.24 | -0.25 | -0.23 | -0.12 | -0.16 | -0.29 |
| Mean Pearson residuals for different clade cells | 0.13 | 0.40 | 0.09 | 0.02 | -0.08 | -0.08 | -0.10 | 0.04 |

* Fisher’s exact p-value for each contingency table computed using a Monte Carlo procedure.
