# Supplementary material for: Heterosubtypic Immunity to Influenza A Virus Infections in Mallards May Explain Existence of Multiple Virus Subtypes
Source: PLoS Pathog. 2013 Jun 20;9(6):e1003443. doi: 10.1371/journal.ppat.1003443 (PMC3688562; doi:10.1371/journal.ppat.1003443)
Supplement: Table S16 — Summary table of the exploration of the contingency tables at the NA subtype level for the short lag. (DOC) [file ppat.1003443.s021.doc]

**Table S16.** Summary table of the exploration of the contingency tables at the NA subtype level for the short lag.

| **Number of most common subtypes considered** | **2** | **3** | **4** | **5** | **6** | **7** | **8** | **9** |
| --- | --- | --- | --- | --- | --- | --- | --- | --- |
| Number of cells | 4 | 9 | 16 | 25 | 36 | 42 | 56 | 81 |
| Number of cells with expected frequency <5 | 4 | 9 | 16 | 25 | 36 | 42 | 56 | 81 |
| Number of individuals | 14 | 28 | 35 | 39 | 41 | 42 | 44 | 46 |
| Number of transitions | 15 | 32 | 40 | 44 | 46 | 47 | 54 | 54 |
| Test for H0: independence on the full table | 1.00 | 0.39 | 0.52 | 0.56 | 0.50 | 0.57 | 0.47* | 0.34* |
| Median p-value over 1000 subsamples with a single transition per individual | 1.00 | 0.55 | 0.57 | 0.62 | 0.58 | 0.59 | 0.66* | 0.53* |
| Mean Pearson residuals for same subtype cells | 0.21 | -0.51 | 0.23 | 0.72 | 0.59 | 0.59 | 0.84 | 0.65 |
| Mean Pearson residuals for different subtype same clade cells |  |  | -0.16 | 0.14 | 0.13 | 0.30 | 0.19 | -0.004 |
| Mean Pearson residuals for different clade cells | -0.21 | 0.25 | -0.05 | -0.23 | -0.13 | -0.17 | -0.17 | -0.05 |

* Fisher’s exact p-value for each contingency table computed using a Monte Carlo procedure.
