# Supplementary material for: Heterosubtypic Immunity to Influenza A Virus Infections in Mallards May Explain Existence of Multiple Virus Subtypes
Source: PLoS Pathog. 2013 Jun 20;9(6):e1003443. doi: 10.1371/journal.ppat.1003443 (PMC3688562; doi:10.1371/journal.ppat.1003443)
Supplement: Table S17 — Summary table of the exploration of the contingency tables at the NA subtype level for the long lag. (DOC) [file ppat.1003443.s022.doc]

**Table S17.** Summary table of the exploration of the contingency tables at the NA subtype level for the long lag.

| **Number of most common subtypes considered** | **2** | **3** | **4** | **5** | **6** | **7** | **8** | **9** |
| --- | --- | --- | --- | --- | --- | --- | --- | --- |
| Number of cells | 4 | 9 | 16 | 25 | 36 | 42 | 56 | 64 |
| Number of cells with expected frequency <5 | 4 | 9 | 16 | 25 | 36 | 42 | 56 | 64 |
| Number of individuals | 6 | 8 | 16 | 21 | 23 | 25 | 27 | 29 |
| Number of transitions | 6 | 10 | 21 | 27 | 29 | 33 | 35 | 38 |
| Test for H0: independence on the full table | 1.00 | 1.00 | 0.91 | 0.74 | 0.66 | 0.74 | 0.83 | 0.40* |
| Median p-value over 1000 subsamples with a single transition per individual |  | 1.00 | 0.87 | 0.76 | 0.72 | 0.79 | 0.86 | 0.66 |
| Mean Pearson residuals for same subtype cells | -1.10 | -0.71 | -0.58 | -0.55 | 0.20 | 0.23 | 0.18 | 0.21 |
| Mean Pearson residuals for different subtype same clade cells |  |  | 0.17 | -0.33 | -0.28 | -0.30 | -0.27 | -0.31 |
| Mean Pearson residuals for different clade cells | 1.10 | 0.35 | 0.20 | 0.24 | 0.01 | 0.03 | 0.02 | 0.02 |

* Fisher’s exact p-value for each contingency table computed using a Monte Carlo procedure.
