# Supplementary material for: Heterosubtypic Immunity to Influenza A Virus Infections in Mallards May Explain Existence of Multiple Virus Subtypes
Source: PLoS Pathog. 2013 Jun 20;9(6):e1003443. doi: 10.1371/journal.ppat.1003443 (PMC3688562; doi:10.1371/journal.ppat.1003443)
Supplement: Table S18 — Summary table of the exploration of the contingency tables at the NA clade level for the whole dataset. (DOC) [file ppat.1003443.s023.doc]

**Table S18.** Summary table of the exploration of the contingency tables at the NA clade level for the whole dataset.

| **Number of most common clades considered** | **2 most common clades** | **3 most common clades** | **All clades** | **Group level-All clades** |
| --- | --- | --- | --- | --- |
| Number of cells | 4 | 9 | 16 | 4 |
| Number of cells with expected frequency <5 | 0 | 2 | 9 | 0 |
| Number of individuals | 44 | 62 | 70 | 70 |
| Number of transitions | 53 | 81 | 92 | 92 |
| Test for H0: independence on the full table | 1.00 | 0.58 | 0.19 | 0.67 |
| Median p-value over 1000 subsamples with a single transition per individual | 1.00 | 0.80 | 0.51 | 0.81 |
| Mean Pearson residuals for same clade cells | -0.16 | -0.60 | 0.22 | -0.48 |
| Mean Pearson residuals for different clade cells | 0.16 | 0.29 | -0.04 | 0.48 |

* Fisher’s exact p-value for each contingency table computed using a Monte Carlo procedure. NA clades are in decreasing frequency order: N3 Clade (N2, N3), N7 Clade (N6, N7, N9), N4 Clade (N1, N4), N8 Clade (N5, N8). The two NA groups in decreasing frequency order are: Group 2 (N3 Clade and N7 Clade) and Group 1 (N4 Clade and N8 Clade).
