# Supplementary material for: Heterosubtypic Immunity to Influenza A Virus Infections in Mallards May Explain Existence of Multiple Virus Subtypes
Source: PLoS Pathog. 2013 Jun 20;9(6):e1003443. doi: 10.1371/journal.ppat.1003443 (PMC3688562; doi:10.1371/journal.ppat.1003443)
Supplement: Table S19 — Summary table of the exploration of the contingency tables at the NA clade level for the short lag. (DOC) [file ppat.1003443.s024.doc]

**Table S19.** Summary table of the exploration of the contingency tables at the NA clade level for the short lag.

| **Number of most common clades considered** | **2 most common clades** | **3 most common clades** | **All clades** | **Group level-All clades** |
| --- | --- | --- | --- | --- |
| Number of cells | 4 | 9 | 16 | 4 |
| Number of cells with expected frequency <5 | 0 | 5 | 12 | 0 |
| Number of individuals | 25 | 42 | 46 | 46 |
| Number of transitions | 27 | 47 | 54 | 54 |
| Test for H0: independence on the full table | 0.26 | 0.29 | 0.17 | 0.39 |
| Median p-value over 1000 subsamples with a single transition per individual | 0.24 | 0.32 | 0.31 | 0.35 |
| Mean Pearson residuals for same clade cells | 1.30 | 0.21 | 0.51 | 1.05 |
| Mean Pearson residuals for different clade cells | -1.30 | -0.11 | -0.16 | -1.05 |

* Fisher’s exact p-value for each contingency table computed using a Monte Carlo procedure. NA clades are in decreasing frequency order: N3 Clade (N2, N3), N7 Clade (N6, N7, N9), N4 Clade (N1, N4), N8 Clade (N5, N8). The two NA groups in decreasing frequency order are: Group 2 (N3 Clade and N7 Clade) and Group 1 (N4 Clade and N8 Clade).
