# Supplementary material for: Heterosubtypic Immunity to Influenza A Virus Infections in Mallards May Explain Existence of Multiple Virus Subtypes
Source: PLoS Pathog. 2013 Jun 20;9(6):e1003443. doi: 10.1371/journal.ppat.1003443 (PMC3688562; doi:10.1371/journal.ppat.1003443)
Supplement: Table S20 — Summary table of the exploration of the contingency tables at the NA clade level for the long lag. (DOC) [file ppat.1003443.s025.doc]

**Table S20.** Summary table of the exploration of the contingency tables at the NA clade level for the long lag.

| **Number of most common clades considered** | **2 most common clades** | **3 most common clades** | **All clades** | **Group level –All clades** |
| --- | --- | --- | --- | --- |
| Number of cells | 4 | 9 | 16 | 4 |
| Number of cells with expected frequency <5 | 0 | 5 | 13 | 0 |
| Number of individuals | 22 | 25 | 29 | 29 |
| Number of transitions | 26 | 34 | 38 | 38 |
| Test for H0: independence on the full table | 0.23 | 0.22 | 0.30 | 0.06 |
| Median p-value over 1000 subsamples with a single transition per individual | 0.39 | 0.26 | 0.33 | 0.14 |
| Mean Pearson residuals for same clade cells | -1.53 | -1.15 | -0.22 | -1.99 |
| Mean Pearson residuals for different clade cells | 1.53 | 0.56 | 0.09 | 1.99 |

* Fisher’s exact p-value for each contingency table computed using a Monte Carlo procedure.
